# The Diagnostic Value of Radiomics-Based Machine Learning in Predicting the Grade of Meningiomas Using Conventional Magnetic Resonance Imaging: A Preliminary Study

**DOI:** 10.3389/fonc.2019.01338

**Published:** 2019-12-06

**Authors:** Chaoyue Chen, Xinyi Guo, Jian Wang, Wen Guo, Xuelei Ma, Jianguo Xu

**Affiliations:** ^1^Department of Biotherapy, Cancer Center, West China Hospital, Sichuan University, Chengdu, China; ^2^State Key Laboratory of Biotherapy and Cancer Center, West China Hospital, Sichuan University, and Collaborative Innovation Center for Biotherapy, Chengdu, China; ^3^Department of Neurosurgery, West China Hospital, Sichuan University, Chengdu, China; ^4^West China School of Medicine, West China Hospital, Sichuan University, Chengdu, China; ^5^School of Computer Science, Nanjing University of Science and Technology, Nanjing, China

**Keywords:** radiomics, machine learning, magnetic resonance imaging, meningioma, tumor grade

## Abstract

**Objective:** The purpose of the current study is to investigate whether texture analysis-based machine learning algorithms could help devise a non-invasive imaging biomarker for accurate classification of meningiomas using machine learning algorithms.

**Method:** The study cohort was established from the hospital database by reviewing the medical records. Patients were selected if they underwent meningioma resection in the neurosurgery department between January 2015 and December 2018. A total number of 40 texture parameters were extracted from pretreatment postcontrast T1-weighted (T1C) images based on six matrixes. Three feature selection methods were adopted, namely, distance correlation, least absolute shrinkage and selection operator (LASSO), and gradient boosting decision tree (GBDT). Multiclass classification methods of linear discriminant analysis (LDA) and support vector machine (SVM) algorithms were employed to establish the classification models. The diagnostic performances of models were evaluated with confusion matrix based on which the areas under the curve, accuracy, and Kappa value of models were calculated.

**Result:** Confusion matrix showed that the LDA-based models represented better diagnostic performances than SVM-based models. The highest accuracy among LDA-based models was 75.6%, shown in the combination of Lasso + LDA. The optimal models for SVM-based models was Lasso+SVM, with accuracy of 59.0% in the testing group. One of the SVM-based models, GBDT+SVM, was overfitting, suggesting that this model was not suitable for application.

**Conclusion:** Machine learning algorithms with texture features extracted from T1C images could potentially serve as the assistant imaging biomarkers for presurgically grading meningiomas.

## Introduction

According to the survey conducted by the Central Brain Tumor Registry of the United States (CBTRUS), meningiomas are one of the most frequent intracranial tumors in adults, with an incidence of 8.14/100,000, accounting for 36.8% of the primary central nervous system tumors (1). In most cases, meningiomas are histologically recognized as low-grade meningioma (WHO grade I) with benign behaviors, but approximately 10–20% of meningiomas are recognized as high-grade meningioma (WHO grades II and III), exhibiting aggressive behaviors (2–4). The treatment and prognosis for meningioma are intimately related to the histopathological grade (5). Surgical resection is the first-line treatment for all types of meningiomas, the extent of surgical resection is the most important prognostic factor for high-grade meningioma outcomes. According to the previous investigations, adjuvant radiotherapy is associated with statistically improved overall survival (OS) and progression-free survival (PFS) outcomes (6–9). Moreover, the prognoses of different grades of meningiomas are dramatically different that higher grades meningiomas are correlated to higher recurrence rate (7–25, 29–52, and 50–94%, respectively) and poor survival outcomes (5, 10). Given these differences in treatment and prognosis, the accurate presurgical assessment on tumor grade is clinically important to facilitate treatment decisions.

Lacking specific blood biomarkers, magnetic resonance imaging (MRI) is the most importent imaging technique in the detection and presurgical assessment of intracranial meningiomas. Previous studies demonstrated that preoperative MRI was useful for assessing the grades and evaluating histopathological characteristics of meningiomas (11–14). However, the image patterns of different grades of meningiomas could mimic each other in some cases, resulting in limited diagnostic accuracy and highlighting the urgency of new radiological evaluation methods (15). Texture analysis is a subset of radiomics. With the ability of mathematically converting medical images into mineable quantitative statistics, it has been considered as the emerging field providing a non-invasive assessment on tumor heterogeneity (16). Theoretically, the texture parameters can objectively calculate the structural and spectral characteristics of pixel intensities within an area to extract quantitative metrics that are impossible to assess visually (17, 18). Compared with traditional visual assessment, texture analysis can describe the image with quantitative statistics more sensitively and accurately (19).

Texture analysis has shown promising diagnostic ability in meningioma grading in previous studies (14, 20–22). Additionally, the quantitative evaluation of texture features has been applied into machine learning technology to differentiate high-grade meningiomas from low-grade meningiomas (20, 21). In the current study, we applied multiple classification methods to systematically grade meningiomas. Six models were established and evaluated, aiming to preliminarily investigate the value of radiomics-based machine learning technology in in preoperative prediction of meningioma grades.

## Materials and Methods

### Patient Selection

This retrospective study was led in the neurosurgery department of our hospital. We viewed the electronic medical records to search for patients with detailed pathological reports on meningiomas between January 2015 and December 2018. The presurgical high-quality MR images of patients were also exported with standard format through PACS (Picture Archiving and Communication System). After the initial evaluation on images and patient profiles, we excluded some patients due to the following reasons: (1) images with motion artifacts; (2) relevant tumor treatment history (like radiotherapy or surgery) in other hospitals; (3) recorded intracranial diseases history, such as subarachnoid hemorrhage, cerebral infarction, and so on. Finally, a total number of 150 meningioma patients were introduced in our study. Clinical information and pathological reports were also recorded for further analysis. It is worth noting that pathological grading was corrected based on the 2016 WHO classification system, adjusted by a senior neuropathologist with 10 years of experience.

The institutional review board approved this retrospective study. All procedures performed in studies involving human participants were in accordance with the ethical standards of the institutional and/or national research committee and with the 1964 Helsinki declaration and its later amendments or comparable ethical standards. The obligatory written informed consent was obtained from participants enrolled in this study (written informed consent for patients <16 years old was signed by parents or guardians). The patients agreed to undertake examination and were informed that the statistics (including MR image), which could be used for academic purpose in the future, would be stored in our institutional database. The Ethics Committee of Sichuan University and neurosurgery department of our institution have given approval for statistics export and utilization for this study.

### MRI Acquisition

**Figure 1:**
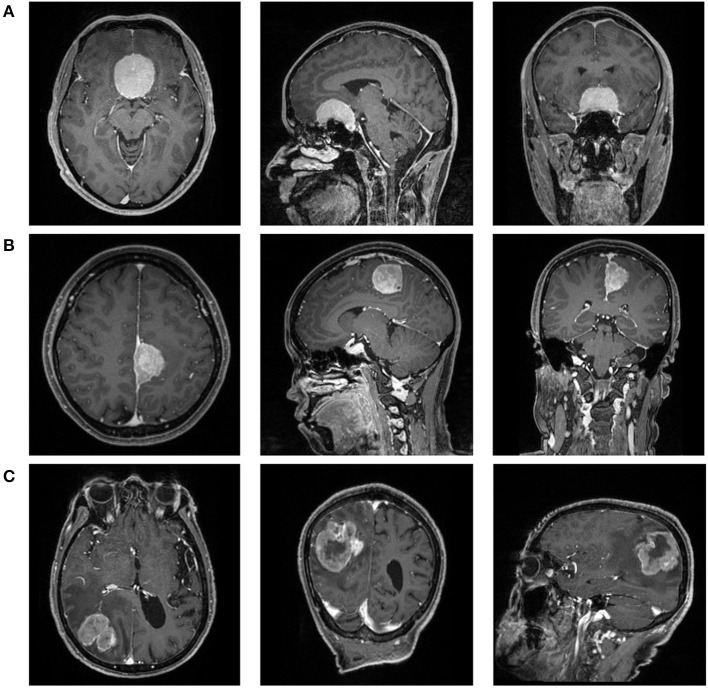
The magnetic resonance images [postcontrast T1-weighted (T1C)] of a patient with **(A)** WHO I meningioma, **(B)** WHO II meningioma, and **(C)** WHO III meningioma.

After consulting with senior radiologists and neurosurgeons, postcontrast T1-weighted (T1C) images were selected for further analysis due to clear depiction of tumor location and boundary (Figure 1). The MR scan was conducted in the MR Research Center of our hospital with 3.0T Siemens Trio Scanner. High-quality three-dimensional T1-weighted images were obtained by using a magnetization prepared rapid gradient-echo (MPRAGE) sequence by the following protocols: TR/TE/TI = 1,900/2.26/900 ms, Flip angle = 9°, 176 axial slices with thickness = 1 mm, axial FOV = 25.6 × 25.6 cm^2^, and data matrix = 256 × 256. The contrast-enhanced image was acquired with gadopentetate dimeglumine (dose: 0.1 mmol/kg) as the contrast agent.

### Texture Features Extraction

The texture analysis was conducted with LIFEx software by two neurosurgeons following the software instructions (23). The authors contoured along the tumor tissue slice by slice to draw the region of interest (ROI), and the three-dimensional texture features were automatically generated with default setting. Any disagreement regarding the tumor location or border of lesions were resolved by consulting senior neurosurgeons and the senior radiologist. Forty quantified texture features were extracted, including features from histogram-based matrix and shape-based matrix from the first order and features from gray-level co-occurrence matrix (GLCM), gray-level zone length matrix (GLZLM), neighborhood gray-level dependence matrix (NGLDM), and gray-level run length matrix (GLRLM) from second or higher order (Supplement Material 1). The definitions of texture parameters were summarized in Supplement Material 2. The association between texture parameters was evaluated using Pearson correlation coefficient test.

### Machine Learning Classification

The classification models were built with different combinations of three selection methods [distance correlation, least absolute shrinkage and selection operator (LASSO), and gradient boosting decision tree (GBDT)] and two multiclass classification algorithms [linear discriminant analysis (LDA) and support vector machine (SVM)]. The feature selection was essential to the diagnostic performance given that diagnostic values on all features were discrepant, and that optimal features can statistically eliminate overfitting. Moreover, it can contribute to decreased running time and increased accuracy of the models. With selected features retrieved from different methods, the statistics were employed into algorithms separately. Two multiple classification algorithms were adopted in the current study, including LDA and SVM, representing the linear classifier and non-linear classifier, respectively. The patients were randomly separated into two parts in the proportion of 4:1 as the training group and the testing group. Confusion matrixes and areas under the curve (AUC) of each model were calculated to evaluate the performance of the models. The algorithms deployment procedure was repeated 100 times to obtain the realistic distribution of classification accuracies.

The regular statistical analyses in this study were conducted using SPSS software (version 21; IBM, Chicago), including Mann-Whitney *U*-test and Pearson correlation coefficient. The machine learning algorithms were programmed using Python Programming Language and scikit-learn package.

## Results

### Characteristics of the Study Cohort

A total number of 150 patients were involved in the current study, among whom 61 were diagnosed with WHO I meningioma, 59 with WHO II meningiomas, and 30 with WHO III meningiomas. The mean ages of patients were 49.38, 54.41, and 56.93 years, respectively. The gender ratio for patients was 62:88 (male:female). The clinical characteristics of patients and tumors were summarized in Table 1.

**Table 1:** Characteristics of patients and lesions.

	**Low-grade meningioma**	**High-grade meningioma**	
	**WHO I meningioma**	**WHO II meningioma**	**WHO III meningioma**
Number	61	59	30
Age	49.38	54.41	56.93
Gender (*n*, %)			
Male	16 (26.23%)	32 (54.24%)	14 (46.67%)
Female	45 (73.77%)	27 (45.76%)	16 (53.33%)
Maximum diameter (cm)	4.06 ± 1.53	5.75 ± 1.50	6.93 ± 2.03
Location (*n*, %)			
Cerebral convexity	32 (52.46%)	40 (67.80%)	21 (70.00%)
Falx	11 (18.03%)	8 (13.56%)	2 (6.67%)
Skull base	18 (29.51%)	11 (18.64%)	7 (23.33%)
Days between MR scan and surgery	8.7 days	7.2 days	6.7 days

### Characteristics of Texture Parameters

**Figure 2:**
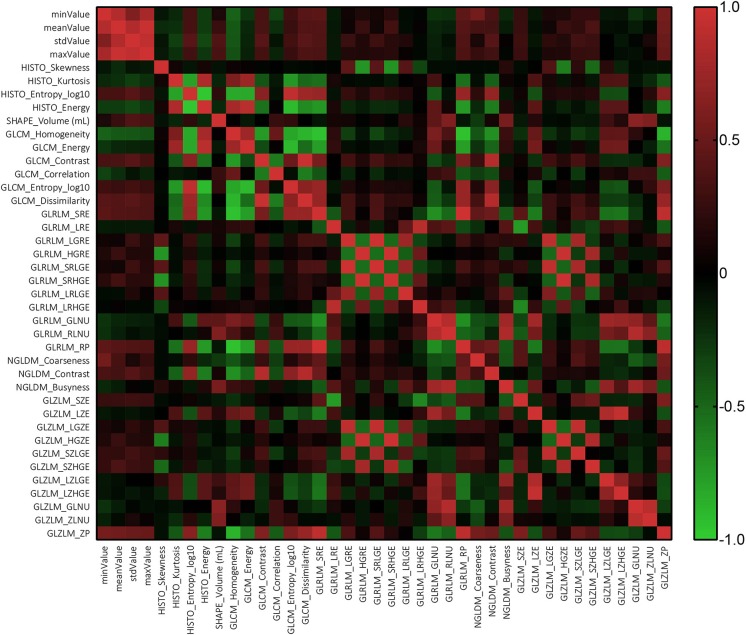
The heat map of relationship among texture analysis parameters.

The results of Mann-Whitney suggested that there was no statistically significant difference among the parameters extracted by two neurosurgeons, implying that the results could be considered reliable and reproducible (Supplement Material 3). The Pearson correlation coefficient suggested that most texture features were correlated with each other rather than independent (Figure 2).

### Diagnostic Performance of Models

In the feature selection, some mutual features were selected when using different methods, suggesting that they were the most significant features in discrimination (Table 2). Generally, the LDA-based models represented better performance than SVM-based models. The accuracy rates for the LDA-based models were 73.0, 75.6, and 73.3 in the testing group whereas for the SVM-based models were 57.6 and 59.0%. Overfitting was observed in one model, SVM+GBDT, suggesting that this model was inappropriate in application. The AUC, Kappa value, and accuracy of each model were represented in Table 3.

**Table 2:** Selected features using distance correlation, LASSO, and GBDT.

**Selection method**	**Selected features**
Distance Correlation	HISTO_Kurtosis, HISTO_Entropy, HISTO_Energy, SHAPE_Volume, GLCM_Energy, GLCM_Entropy_log10, NGLDM_Contrast, GLZLM_ZLNU
LASSO	minValue, meanValue, stdValue, SHAPE_Volume (ml), GLCM_Contrast, GLRLM_HGRE, GLRLM_LRHGE, GLRLM_GLNU, GLRLM_RLNU, GLZLM_LZE, GLZLM_HGZE, GLZLM_SZHGE, GLZLM_LZHGE, GLZLM_GLNU, GLZLM_ZLNU
GBDT	minValue, HISTO_Skewness, SHAPE_Volume (ml), GLCM_Homogeneity, GLCM_Energy, GLCM_Correlation, GLCM_Entropy_log10, GLCM_Dissimilarity, GLRLM_LRLGE, GLRLM_RLNU, NGLDM_Contrast, GLZLM_SZE, GLZLM_LZHGE, GLZLM_ZLNU

*LASSO, least absolute shrinkage and selection operator; GBDT, gradient boosting decision tree*.

**Table 3:** Diagnostic performance of classification models.

**Models**		**Training group**				**Validation group**			
		**WHO Grade I**	**WHO Grade II**	**WHO Grade III**	**Kappa value**	**WHO Grade I**	**WHO Grade II**	**WHO Grade III**	**Kappa value**
LDA	Distance Correlation	0.928	0.865	0.882	0.578 (Accuracy = 75.4%)	0.884	0.820	0.846	0.563 (Accuracy = 73.0%)
	LASSO	0.955	0.914	0.915	0.693 (Accuracy = 80.8%)	0.934	0.846	0.783	0.603 (Accuracy = 75.6%)
	GBDT	0.928	0.950	0.908	0.570 (Accuracy = 73%)	0.886	0.854	0.887	0.572 (Accuracy = 73.3%)
SVM	Distance Correlation	0.870	0.831	0.876	0.356 (Accuracy = 61.1%)	0.845	0.798	0.845	0.274 (Accuracy = 57.6%)
	LASSO	0.898	0.806	0.877	0.373 (Accuracy = 62.0%)	0.840	0.772	0.833	0.298 (Accuracy = 59.0%)
	GBDT (Overfitting)	–	–	–	–	–	–	–	

*LDA, Linear Discriminate Analysis; SVM, Support Vector Machine; LASSO, Least absolute shrinkage and selection operator; GBDT, gradient boosting decision tree*.

**Figure 3:**
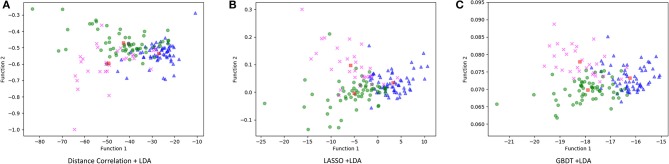
Distribution of the discriminant functions of LDA models. **(A)** Distance correlation + LDA; **(B)** least absolute shrinkage and selection operator (LASSO) + LDA; and **(C)** gradient boosting decision tree (GBDT) + LDA.

**Figure 4:**
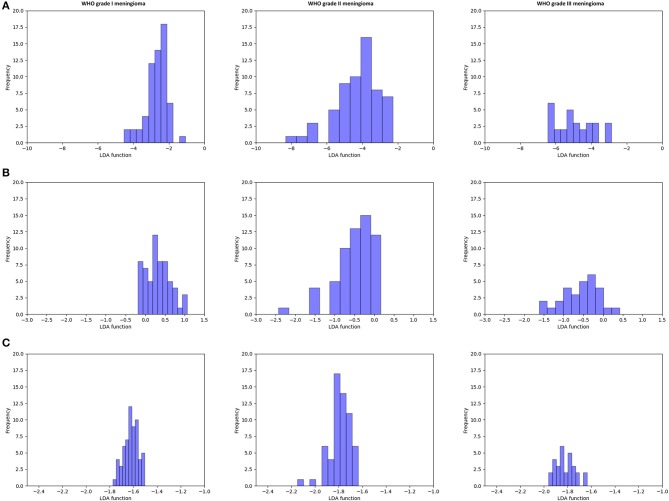
Example of distributions of the linear discriminant analysis (LDA)-based models determined for the lesions for one cycle. **(A)** Distance correlation + LDA; **(B)** least absolute shrinkage and selection operator (LASSO) + LDA; and **(C)** gradient boosting decision tree (GBDT) + LDA.

Figure 3 illustrated the performance of the LDA-based models in terms of the distribution of the canonical functions for one of the 100 independent training cycles. Figure 4 illustrated the examples of distributions of the LDA function determined for the lesions for one cycle.

## Discussion

The prediction of the histopathological meningioma grade is important because it is closely related to survival outcomes and treatment strategies. According to the instructions of the National Comprehensive Cancer Network (NCCN) guideline, the recommended treatment for WHO grade I meningioma was surgical resection or observation; for WHO grade II meningioma, it was gross total resection combined with/without radiotherapy; and for a WHO grade III meningioma, it was radical surgery with radiotherapy (24). Therefore, the accurate preoperative diagnosis should assist clinicians in making a personalized treatment plan to improve the quality of life. In the current study, we investigated the diagnostic value of texture analysis-based machine learning technology in meningioma grade. The texture features adopted into the classifiers were extracted from T1C images, which brought the possibility to utilize the technology in standard routine care imaging analyses.

Texture analysis provides information on the heterogeneity of tumor imaging, such as tumor cellularity, degenerative changes, and neovascularization, which are hard to assess visually. By analyzing the spectral distribution of pixels, abnormal tumor microenvironment and pathology could be represented as a series of statistics (25). It has been reported that an imaging technology extends beyond radiology to histopathology, like prediction on gene mutation and tumor grading (26–32). As for the different grade meningiomas, the characteristics of enhanced pattern have been reported in previous researches. Specifically, MRI features, such as positive capsular enhancement, indistinct tumor–brain interface, and heterogeneous tumor enhancement, were suggested to be related to a higher tumor grade (33, 34). These MRI features could be reflected in GLZLM_ZLNU, one of the mutual selected features in our study. This feature calculates the non-uniformity of the gray-levels or the length of the homogeneous zones, reflecting the heterogeneity within the delineated area. Fluctuance on value of features from second or higher order represented irregular changes in the gray pixels of aggressive meningiomas due to the heterogeneous structure inside tumor tissue (11). Therefore, it is reasonable to consider that this MRI feature was closely correlated to this texture feature. Another mutual feature, SHAPE_Volume (ml), suggested that the tumor volume was also in relation to grade, according to the differences in tumor diameter. However, it is worthy to note that most features were correlated with each other; the specific reason is still unclear that is why GLZLM_ZLNU was selected as the strongest correlated feature while others were not. Future researches are required to explore this question.

The value of radiomics-based machine learning in meningioma grading has been explored before. Retrieved parameters, feature selection method, sample size, and classification algorithms determined the performance of models. However, all of these studies, as well as this study, was seriously limited by the small sample size due to the rather low incidents of grade III meningioma. Therefore, most of them simply classified them into low-grade and high-grade (21, 35). Only one study explored the multiple classification models in discrimination, which established models with the parameters extracted from ADC map and decision trees algorithms, demonstrating the equivalent diagnostic performance of machine learning technology compared to experienced neuroradiologists (accuracy = 79.51%, Kappa value = 0.6393) (14). As for this study, we employed different multiple classification algorithms and texture features from different sequences. However, we should note that the differences between the models were not strong enough to select the optimal one, specifically considering that the investigated models seemed to perform quite comparably and that the variance in AUC might be partially attributed to the small sample size. Therefore, our results could only be regarded as a hypothesis and need to be verified in future studies.

LDA and SVM were employed as classification algorithms in the current study. Both of them are considered state-of-the-art in pattern recognition technology, representing two different types of classifiers (36). LDA is the linear classifier, consisting of the shape of the decision boundary of straight line in the first case and straight line in second, whereas SVM is the non-linear classifier, of which the shape of the decision boundary is a plane in the first case and a plane in the second (36, 37). Computational time and complexity usually increase together when trying to improve the performance. Therefore, the importance on the trade-off between computational burden and performance has been highlighted to require a suitable selection method. Previous studies performed feature selection with Friedman test or Mann-Whitney *U*-test to choose the most significant features into classifiers, suggesting that the selected features could improve the classifier performances (11, 14, 35). The results of our study showed that all LDA-based models represented better performances than SVM-based models, and that the improvement using different selected models was limited. It seemed that the algorithms have more priority than the selection method in the improvement of diagnostic performances. Therefore, in futures studies, researchers should focus on the algorithm selection, and novel algorithms should be investigated.

There were some limitations in the current study. First, this is a single-institution retrospective study that enrolled 150 patients. The patient sample was relatively small, and the selection bias was inevitable. Second, the texture features into classifiers were extracted from T1C sequence, while the value of features from other sequences was unclear. Given that the research using parameters extracted from ADC images represented better performance, future researches were required to investigate whether the diagnostic performance could be improved when combined with features from other sequences and advanced MR technology. Third, novel radiomics parameters have been identified in recent years, while our studies only involved traditional texture parameters. Compared to many other studies in the same field, the number of radiomics features (*n* = 40) is fairly small. Fourth, we did not perform comparison to the performance of a human reader with classifiers. Fifth, only few classification algorithms were evaluated in our study. Machine learning has been developed rapidly in recent years, and new algorithms are being programmed. Sixth, we did not verify the efficacy of machine learning-based models in external datasets. We tried to search the public datasets, but all of them were for gliomas. The software used to extract texture parameters and package to perform machine learning is the open-source package, providing a potential for other researchers to reproduce our researches.

## Data Availability Statement

The datasets generated for this study are available on request to the corresponding author.

## Ethics Statement

The studies involving human participants were reviewed and approved by The Ethics Committee of Sichuan University. Written informed consent to participate in this study was provided by the participants' legal guardian/next of kin.

## Author Contributions

CC participated in collecting MR images, extracting statistics, and drafting the manuscript. XG participated in collecting MR images and extracting statistics. JW established and deployed the machine learning models. WG participated in collecting MR images and extracting statistics. XM and JX participated in conceptualization and revised some intellectual content in the manuscript.

### Conflict of Interest

The authors declare that the research was conducted in the absence of any commercial or financial relationships that could be construed as a potential conflict of interest.

## Supplementary Material

Supplement Material 1 The texture analysis parameters extracted from images.

Supplement Material 2 The definitions of texture analysis parameters.

Supplement Material 3 The results of Mann-Whitney *U*-test in the examination of repeatability of texture analysis parameters extraction.
