# Supplementary material for: The Diagnostic Value of Radiomics-Based Machine Learning in Predicting the Grade of Meningiomas Using Conventional Magnetic Resonance Imaging: A Preliminary Study
Source: Front Oncol. 2019 Dec 6;9:1338. doi: 10.3389/fonc.2019.01338 (PMC6908490; doi:10.3389/fonc.2019.01338)
Supplement: Supplement Material 1 — The texture analysis parameters extracted from images. [file Data_Sheet_1.PDF]

Patient	1	2	3	4	5	6	7	8	9	10	11
Type (grade1=1, grade2=2, grade3=3)	1	1	1	1	1	1	1	1	1	1	1
minValue	141	171	137	5	276	369	181	29	169	167	204
meanValue	646	361	367	421	652	722	872	547	498	601	796
stdValue	65.4	36.1	53.7	61.7	75.3	72.2	169	99.4	45.6	97.2	87.8
maxValue	1270	645	583	855	1070	1080	1330	934	840	1390	1220
HISTO_Skewness	-0.31	0.0961	-0.374	-0.143	0.0405	-0.51	-0.0712	-1.14	0.0733	0.66	-0.0956
HISTO_Kurtosis	11.8	6.94	3.63	6.06	5.42	6.52	3.02	6.45	4.44	6.67	6.27
HISTO_Entropy_log10	1.12	1.27	1.49	1.26	1.37	1.38	1.4	1.38	1.25	1.28	1.33
HISTO_Energy	0.104	0.0686	0.0392	0.0734	0.056	0.056	0.0463	0.0579	0.0682	0.0706	0.0617
SHAPE_Volume (mL)	12.9	1.1	1.5	4.8	0.877	1.5	5.51	4.92	8.37	5.95	4.96
GLCM_Homogeneity	0.519	0.408	0.331	0.443	0.341	0.418	0.354	0.325	0.424	0.422	0.414
GLCM_Energy	0.0214	0.00846	0.00339	0.0103	0.00589	0.00706	0.00376	0.00476	0.00833	0.00962	0.00775
GLCM_Contrast	9.63	17.3	42.5	13.2	33.8	28.5	21.9	41.2	13.1	16.4	16.4
GLCM_Correlation	0.562	0.556	0.564	0.635	0.376	0.62	0.681	0.58	0.609	0.647	0.665
GLCM_Entropy_log10	1.97	2.29	2.69	2.26	2.47	2.44	2.57	2.59	2.28	2.32	2.36
GLCM_Dissimilarity	1.91	2.9	4.57	2.51	4.14	3.24	3.47	4.42	2.61	2.81	2.84
GLRLM_SRE	0.836	0.908	0.935	0.887	0.935	0.904	0.937	0.939	0.894	0.895	0.899
GLRLM_LRE	2.61	1.6	1.39	1.83	1.39	1.65	1.3	1.29	1.71	1.78	1.69
GLRLM_LGRE	0.00137	0.00229	0.00138	0.00118	0.0015	0.00253	0.00163	0.00115	0.00108	0.00233	0.000828
GLRLM_HGRE	862	709	1170	1030	982	1080	817	1430	1040	564	1460
GLRLM_SRLGE	0.00118	0.00214	0.00132	0.00106	0.00143	0.00233	0.00154	0.0011	0.000967	0.00211	0.000756
GLRLM_SRHGE	715	644	1090	909	921	975	765	1350	933	507	1310
GLRLM_LRLGE	0.00322	0.00326	0.00176	0.00201	0.00195	0.00394	0.00204	0.00142	0.00182	0.00402	0.00131
GLRLM_LRHGE	2300	1130	1660	1910	1360	1790	1060	1830	1750	980	2490
GLRLM_GLNU	4420	395	327	1810	274	437	236	259	2990	1700	1610
GLRLM_RLNU	31900	4800	7280	19700	4340	6450	4390	3940	34800	19900	21300
GLRLM_RP	0.777	0.873	0.91	0.843	0.91	0.868	0.916	0.919	0.855	0.855	0.861
NGLDM_Coarseness	0.000154	0.00137	0.00108	0.000307	0.00138	0.0011	0.00188	0.00168	0.000176	0.000314	0.000309
NGLDM_Contrast	0.00941	0.0343	0.0928	0.0227	0.0676	0.0535	0.102	0.0857	0.0223	0.0277	0.0306
NGLDM_Busyness	2	0.372	0.329	1.19	0.33	0.321	0.434	0.217	2.19	1.65	0.908
GLZLM_SZE	0.604	0.622	0.592	0.588	0.645	0.597	0.665	0.709	0.546	0.535	0.557
GLZLM_LZE	46900	501	80.2	5570	105	766	64.5	112	7030	4890	3170
GLZLM_LGZE	0.00253	0.00405	0.00215	0.00167	0.00216	0.00362	0.0021	0.0016	0.00134	0.00275	0.00132
GLZLM_HGZE	750	717	1060	941	1010	1020	766	1500	1080	650	1330
GLZLM_SZLGE	0.0019	0.00275	0.00164	0.000998	0.00171	0.00207	0.00159	0.00133	0.000853	0.00136	0.000937
GLZLM_SZHGE	439	449	598	534	644	594	491	1070	582	378	692
GLZLM_LZLGE	52.5	0.715	0.068	5.56	0.119	0.818	0.0908	0.0873	7.31	9.3	2.22
GLZLM_LZHGE	42200000	366000	105000	5640000	96200	782000	51800	153000	6860000	2610000	4590000
GLZLM_GLNU	261	53.2	83.8	192	63.4	56.5	69.9	63.4	304	150	173
GLZLM_ZLNU	1850	482	891	1430	667	595	750	782	1880	1090	1360
GLZLM_ZP	0.0924	0.196	0.291	0.145	0.311	0.192	0.326	0.334	0.127	0.136	0.15

Patient	12	13	14	15	16	17	18	19	20	21	22
Type (grade1=1, grade2=2, grade3=3)	1	1	1	1	1	1	1	1	1	1	1
minValue	25	219	141	179	41	1550	199	25	149	261	188
meanValue	671	711	563	354	226	2560	410	897	585	537	345
stdValue	70.4	49.4	76.9	43.7	27.9	316	52.7	111	64.6	47.6	46.7
maxValue	1260	999	1060	643	387	3310	838	1430	896	963	729
HISTO_Skewness	-0.18	-0.837	0.0907	-0.457	0.152	-0.968	1.19	-0.385	-0.627	1.02	1.45
HISTO_Kurtosis	11.2	8.46	6.2	4.14	6.49	3.58	7.79	5.36	5.33	8.92	7.04
HISTO_Entropy_log10	1.11	1.18	1.32	1.35	1.29	1.6	1.28	1.31	1.34	1.19	1.3
HISTO_Energy	0.111	0.0868	0.0613	0.0552	0.067	0.0307	0.071	0.059	0.056	0.0892	0.0643
SHAPE_Volume (mL)	5.55	7.87	4.08	3.9	4.52	2.76	2.26	3.27	21.4	5.64	13.2
GLCM_Homogeneity	0.524	0.447	0.379	0.302	0.461	0.209	0.406	0.466	0.396	0.483	0.413
GLCM_Energy	0.0236	0.0129	0.00647	0.00487	0.0102	0.00262	0.00923	0.0087	0.00562	0.0161	0.00883
GLCM_Contrast	8.8	13.4	23.3	52	13	174	21.8	14.1	14.4	10.1	24.7
GLCM_Correlation	0.582	0.487	0.568	0.144	0.704	0.274	0.532	0.694	0.727	0.661	0.495
GLCM_Entropy_log10	1.94	2.15	2.43	2.5	2.27	2.65	2.33	2.3	2.44	2.09	2.35
GLCM_Dissimilarity	1.86	2.48	3.37	5.22	2.38	9.66	3.13	2.42	2.79	2.12	3.24
GLRLM_SRE	0.833	0.88	0.92	0.95	0.873	0.976	0.907	0.869	0.912	0.86	0.895
GLRLM_LRE	2.59	1.87	1.47	1.25	1.97	1.11	1.61	2.14	1.44	2.18	1.91
GLRLM_LGRE	0.00101	0.000681	0.00148	0.00282	0.000998	0.00367	0.00299	0.000747	0.000894	0.00177	0.00353
GLRLM_HGRE	1170	1680	934	641	1230	1520	502	1650	1460	686	407
GLRLM_SRLGE	0.000867	0.000607	0.00138	0.00271	0.000889	0.00364	0.00275	0.000663	0.000831	0.00154	0.00314
GLRLM_SRHGE	972	1470	861	606	1080	1480	459	1430	1330	595	371
GLRLM_LRLGE	0.00238	0.00121	0.00204	0.00334	0.00183	0.00379	0.00457	0.00147	0.00121	0.00373	0.00699
GLRLM_LRHGE	3060	3170	1370	818	2430	1710	775	3520	2120	1450	701
GLRLM_GLNU	2690	3400	302	189	1490	21.2	830	995	1050	2140	2880
GLRLM_RLNU	18400	31000	4200	3130	17400	657	9900	13000	15300	18800	37300
GLRLM_RP	0.773	0.836	0.89	0.933	0.826	0.968	0.871	0.82	0.882	0.808	0.85
NGLDM_Coarseness	0.000246	0.000161	0.00156	0.00167	0.000385	0.00961	0.000621	0.000539	0.000486	0.000284	0.000146
NGLDM_Contrast	0.0101	0.0175	0.0397	0.112	0.0239	0.472	0.0353	0.0246	0.0333	0.0167	0.0431
NGLDM_Busyness	1.2	1.77	0.266	0.372	0.836	0.0418	0.89	0.56	0.62	1.52	4.26
GLZLM_SZE	0.588	0.582	0.611	0.664	0.582	0.771	0.604	0.58	0.703	0.554	0.56
GLZLM_LZE	33900	18300	285	30.1	7520	3.83	1440	4900	2740	19500	6880
GLZLM_LGZE	0.00207	0.000963	0.00218	0.00337	0.00174	0.00482	0.00397	0.00142	0.00148	0.00236	0.00318
GLZLM_HGZE	1050	1550	969	596	1130	1450	602	1540	1400	791	584
GLZLM_SZLGE	0.00163	0.000557	0.00166	0.00214	0.00127	0.00444	0.00227	0.00111	0.00123	0.00128	0.00161
GLZLM_SZHGE	595	860	604	388	609	1080	383	846	975	452	354
GLZLM_LZLGE	28.7	10.6	0.321	0.0465	6.36	0.00758	3.42	3.11	1.87	32.6	26.7
GLZLM_LZHGE	40200000	31700000	258000	22800	9040000	6340	617000	7910000	4080000	11800000	1820000
GLZLM_GLNU	138	254	57.6	66.6	126	12.7	102	90.4	153	125	333
GLZLM_ZLNU	936	1880	497	659	1080	261	950	734	1980	912	2470
GLZLM_ZP	0.0841	0.121	0.249	0.426	0.12	0.654	0.197	0.111	0.199	0.0988	0.151

Patient	23	24	25	26	27	28	29	30	31	32	33
Type (grade1=1, grade2=2, grade3=3)	1	1	1	1	1	1	1	1	1	1	1
minValue	113	226	1280	335	86	46	282	201	196	424	166
meanValue	675	551	2780	624	644	915	741	476	694	793	545
stdValue	93.2	55.2	351	80	120	202	96.7	59.6	60	161	39.8
maxValue	1320	905	4160	1080	1020	1740	1300	816	1020	1690	672
HISTO_Skewness	-0.394	0.436	0.0478	0.519	-0.647	-0.397	-0.126	0.638	-0.605	1.97	-1.71
HISTO_Kurtosis	7.34	5	4.91	4.68	4.53	3.41	4.9	7.37	8.31	10.4	12.5
HISTO_Entropy_log10	1.27	1.32	1.45	1.44	1.51	1.49	1.39	1.36	1.25	1.38	1.26
HISTO_Energy	0.068	0.059	0.0508	0.0446	0.0381	0.0382	0.0501	0.06	0.0709	0.0625	0.0698
SHAPE_Volume (mL)	6.36	3.9	4.17	2.06	12.7	13.5	4.66	2.3	4.1	2.4	2.68
GLCM_Homogeneity	0.417	0.385	0.33	0.314	0.277	0.401	0.308	0.389	0.438	0.335	0.417
GLCM_Energy	0.00806	0.00628	0.00577	0.00347	0.00206	0.00371	0.00347	0.00702	0.00961	0.00739	0.00895
GLCM_Contrast	13.5	20	66.5	44.1	56.3	18.5	33.4	26	14.8	77.1	21.6
GLCM_Correlation	0.647	0.583	0.21	0.488	0.568	0.817	0.481	0.585	0.596	0.321	0.448
GLCM_Entropy_log10	2.29	2.41	2.53	2.67	2.88	2.61	2.61	2.43	2.27	2.42	2.29
GLCM_Dissimilarity	2.58	3.23	5.35	4.85	5.47	2.96	4.33	3.41	2.59	5.52	2.93
GLRLM_SRE	0.908	0.914	0.941	0.939	0.953	0.914	0.946	0.912	0.888	0.935	0.9
GLRLM_LRE	1.47	1.55	1.35	1.36	1.21	1.45	1.24	1.61	1.81	1.51	1.68
GLRLM_LGRE	0.00157	0.0012	0.0017	0.00232	0.00124	0.00135	0.00197	0.00167	0.000805	0.00749	0.000641
GLRLM_HGRE	939	1000	1210	693	1570	1160	908	886	1560	443	2370
GLRLM_SRLGE	0.00147	0.0011	0.00163	0.00219	0.0012	0.00127	0.00189	0.00155	0.00073	0.00711	0.000599
GLRLM_SRHGE	851	915	1140	655	1500	1060	861	808	1380	419	2130
GLRLM_LRLGE	0.00209	0.00181	0.00211	0.00308	0.0014	0.00179	0.0023	0.00245	0.00134	0.0104	0.000929
GLRLM_LRHGE	1390	1540	1620	923	1910	1710	1120	1420	2810	619	4000
GLRLM_GLNU	376	921	185	503	498	464	219	733	1560	117	968
GLRLM_RLNU	4510	13100	3320	9940	11700	9830	3850	10300	17600	1700	11400
GLRLM_RP	0.877	0.882	0.919	0.916	0.938	0.883	0.929	0.878	0.846	0.905	0.862
NGLDM_Coarseness	0.00151	0.000471	0.00206	0.000619	0.00068	0.00112	0.00169	0.000631	0.000358	0.00258	0.000513
NGLDM_Contrast	0.0268	0.0381	0.114	0.0824	0.116	0.0597	0.0778	0.0453	0.0201	0.133	0.0305
NGLDM_Busyness	0.247	1	0.185	0.742	0.451	0.325	0.28	0.569	0.703	0.246	0.448
GLZLM_SZE	0.73	0.573	0.638	0.592	0.664	0.712	0.677	0.58	0.587	0.615	0.616
GLZLM_LZE	1050	758	62.1	52.8	23.4	477	51.3	650	4870	93.6	1830
GLZLM_LGZE	0.00304	0.0015	0.00222	0.00268	0.00178	0.00227	0.00288	0.0027	0.00149	0.00988	0.00145
GLZLM_HGZE	889	1020	1250	765	1540	1030	945	894	1510	597	2200
GLZLM_SZLGE	0.00268	0.00101	0.0013	0.00171	0.00146	0.0019	0.00236	0.00199	0.00119	0.0069	0.00126
GLZLM_SZHGE	644	590	791	467	1000	709	649	505	848	432	1290
GLZLM_LZLGE	1.07	0.831	0.0608	0.107	0.0172	0.433	0.0684	0.803	3.33	0.404	0.789
GLZLM_LZHGE	1050000	710000	69600	30800	36900	589000	42300	541000	7180000	30200	4290000
GLZLM_GLNU	52.9	155	44.7	139	182	102	67.9	99.3	149	22.1	120
GLZLM_ZLNU	668	1120	546	1280	2440	1630	740	878	1200	219	1030
GLZLM_ZP	0.206	0.201	0.349	0.312	0.423	0.25	0.366	0.194	0.138	0.289	0.175

Patient	34	35	36	37	38	39	40	41	42	43	44
Type (grade1=1, grade2=2, grade3=3)	1	1	1	1	1	1	1	1	1	1	1
minValue	234	78	497	167	315	78	710	51	277	207	439
meanValue	667	412	908	520	931	203	5000	197	853	1300	1380
stdValue	66.5	58.1	163	70.9	198	46.6	1320	60.5	136	145	132
maxValue	995	604	1600	944	2070	643	7230	582	1310	2340	2170
HISTO_Skewness	-1.15	-0.787	0.158	0.257	0.442	2.34	-0.751	1.08	-0.422	-1.12	-0.317
HISTO_Kurtosis	9.2	4.48	2.96	4.67	3.92	13.8	3.36	4.83	3.33	8.58	5.52
HISTO_Entropy_log10	1.31	1.44	1.57	1.37	1.46	1.24	1.55	1.43	1.53	1.18	1.29
HISTO_Energy	0.0641	0.0436	0.0307	0.0525	0.0416	0.0781	0.0359	0.0473	0.0344	0.0906	0.0648
SHAPE_Volume (mL)	12.9	11.5	4.59	3.74	9.9	0.529	0.465	6.52	11.9	28.1	23.8
GLCM_Homogeneity	0.364	0.33	0.303	0.347	0.312	0.399	0.226	0.319	0.282	0.484	0.401
GLCM_Energy	0.00624	0.00362	0.00248	0.00501	0.00335	0.0107	0.0181	0.00482	0.00217	0.0181	0.00798
GLCM_Contrast	29.7	40.3	106	29.4	54.9	27.4	195	68.3	72.6	15	22.6
GLCM_Correlation	0.388	0.49	0.378	0.496	0.426	0.32	0.24	0.288	0.42	0.495	0.436
GLCM_Entropy_log10	2.44	2.65	2.81	2.52	2.69	2.21	1.85	2.56	2.85	2.1	2.35
GLCM_Dissimilarity	3.69	4.46	6.8	3.96	5.33	3.31	9.59	5.53	6.04	2.4	3.27
GLRLM_SRE	0.925	0.934	0.943	0.928	0.939	0.916	0.983	0.941	0.952	0.845	0.899
GLRLM_LRE	1.42	1.38	1.35	1.45	1.38	1.51	1.07	1.38	1.26	3.05	1.75
GLRLM_LGRE	0.00117	0.000862	0.0049	0.00142	0.00325	0.00703	0.00895	0.00653	0.00131	0.00112	0.001
GLRLM_HGRE	1400	1740	684	910	581	252	1980	392	1370	1100	1270
GLRLM_SRLGE	0.00111	0.000816	0.00471	0.00133	0.00308	0.00647	0.00893	0.00608	0.00126	0.000974	0.000917
GLRLM_SRHGE	1290	1620	644	844	547	236	1950	375	1310	923	1140
GLRLM_LRLGE	0.00155	0.00111	0.00612	0.00198	0.00431	0.0104	0.00901	0.00917	0.00157	0.00297	0.00162
GLRLM_LRHGE	1970	2430	907	1310	789	349	2130	497	1740	3470	2210
GLRLM_GLNU	997	487	112	1080	325	217	4.36	204	278	7310	1070
GLRLM_RLNU	13300	9750	3250	17700	6910	2380	120	3920	7290	64800	13800
GLRLM_RP	0.898	0.91	0.92	0.9	0.915	0.885	0.978	0.918	0.934	0.786	0.864
NGLDM_Coarseness	0.000449	0.000704	0.0021	0.000332	0.000934	0.00232	0	0.00157	0.000945	0.0000672	0.000458
NGLDM_Contrast	0.041	0.0934	0.25	0.0472	0.109	0.0526	0	0.124	0.15	0.0182	0.0349
NGLDM_Busyness	0.61	0.51	0.307	1.08	0.634	0.42	0	0.569	0.388	4.21	0.836
GLZLM_SZE	0.624	0.629	0.598	0.567	0.588	0.636	0.854	0.647	0.661	0.557	0.566
GLZLM_LZE	218	68	32.7	153	48.1	198	2.26	50.4	20.3	73500	957
GLZLM_LGZE	0.00162	0.0011	0.00661	0.00186	0.00394	0.00753	0.011	0.00574	0.00155	0.00203	0.00157
GLZLM_HGZE	1410	1610	711	923	629	378	1960	522	1350	900	1250
GLZLM_SZLGE	0.00106	0.000653	0.00428	0.00127	0.00237	0.00401	0.0109	0.00326	0.001	0.00122	0.00117
GLZLM_SZHGE	877	969	436	522	391	283	1670	377	876	473	699
GLZLM_LZLGE	0.165	0.0386	0.093	0.187	0.111	1.29	0.0121	0.304	0.0182	62.7	0.794
GLZLM_LZHGE	300000	129000	21800	132000	27400	32900	4570	11500	28000	86700000	1170000
GLZLM_GLNU	187	130	36.2	213	88.4	34.3	2.84	58.8	112	396	150
GLZLM_ZLNU	1680	1460	457	1730	852	302	68.1	668	1510	2670	1100
GLZLM_ZP	0.261	0.318	0.339	0.246	0.305	0.245	0.773	0.355	0.426	0.0834	0.184

Patient	45	46	47	48	49	50	51	52	53	54	55
Type (grade1=1, grade2=2, grade3=3)	1	1	1	1	1	1	1	1	1	1	1
minValue	1510	88	75	220	170	3040	199	143	140	31	343
meanValue	3420	449	640	695	677	5100	727	703	560	513	749
stdValue	371	47.4	120	111	69.7	428	83.1	97.6	65	59.2	87.9
maxValue	5100	808	1670	1250	1020	6580	1870	1490	965	946	1220
HISTO_Skewness	-0.569	-0.51	0.602	0.329	-0.395	-0.431	0.534	-0.0235	0.3	-0.206	0.156
HISTO_Kurtosis	6.67	5.66	4.18	3.77	7.16	5.44	15.9	4.6	5.69	6.75	4.21
HISTO_Entropy_log10	1.36	1.22	1.28	1.45	1.3	1.46	1.08	1.27	1.3	1.21	1.41
HISTO_Energy	0.0598	0.0755	0.0616	0.0427	0.0663	0.042	0.107	0.067	0.0647	0.0786	0.0501
SHAPE_Volume (mL)	3.96	9.74	9.1	4.87	4.63	3.15	6.68	19	3.42	6.76	7
GLCM_Homogeneity	0.299	0.469	0.443	0.253	0.442	0.241	0.518	0.412	0.421	0.439	0.38
GLCM_Energy	0.00581	0.0122	0.00904	0.00226	0.00952	0.0036	0.0204	0.00834	0.00824	0.0108	0.00545
GLCM_Contrast	58.5	12.3	13.1	68.8	17.4	68.7	7.8	16.8	16.2	12	27
GLCM_Correlation	0.181	0.594	0.7	0.254	0.617	0.278	0.552	0.553	0.625	0.579	0.634
GLCM_Entropy_log10	2.41	2.17	2.28	2.81	2.3	2.54	1.94	2.32	2.33	2.2	2.54
GLCM_Dissimilarity	5.27	2.34	2.58	6.27	2.69	6.42	1.8	2.9	2.8	2.46	3.57
GLRLM_SRE	0.959	0.862	0.872	0.962	0.878	0.971	0.841	0.897	0.895	0.887	0.911
GLRLM_LRE	1.18	2.25	2.21	1.18	1.96	1.14	2.35	1.73	1.76	1.81	1.61
GLRLM_LGRE	0.00207	0.00106	0.0022	0.00158	0.000838	0.00235	0.00265	0.00158	0.00111	0.00097	0.00148
GLRLM_HGRE	1240	1080	562	959	1530	1500	440	757	1120	1190	960
GLRLM_SRLGE	0.00203	0.000928	0.00192	0.00151	0.00075	0.00233	0.00227	0.00143	0.001	0.000869	0.00137
GLRLM_SRHGE	1190	924	493	925	1340	1450	369	679	1000	1050	876
GLRLM_LRLGE	0.00222	0.00225	0.00481	0.00189	0.00152	0.00246	0.00588	0.00266	0.00184	0.00169	0.00224
GLRLM_LRHGE	1480	2440	1210	1120	2990	1700	1040	1310	1960	2150	1530
GLRLM_GLNU	59.3	2410	1900	638	1530	38.3	3310	4450	1160	2700	1480
GLRLM_RLNU	922	24700	24400	13700	18400	858	22700	53900	14500	27300	24900
GLRLM_RP	0.946	0.815	0.829	0.949	0.835	0.961	0.786	0.859	0.855	0.845	0.877
NGLDM_Coarseness	0.00598	0.000219	0.000258	0.000439	0.000325	0.00701	0.00022	0.00011	0.000404	0.000218	0.000273
NGLDM_Contrast	0.127	0.0187	0.0278	0.106	0.0274	0.154	0.00729	0.0246	0.0325	0.0166	0.0518
NGLDM_Busyness	0.0905	1.64	2.16	0.978	0.823	0.0662	2.1	3.54	1.06	1.53	1.32
GLZLM_SZE	0.703	0.522	0.451	0.684	0.552	0.756	0.533	0.51	0.549	0.571	0.566
GLZLM_LZE	14.4	18000	6800	9.88	7880	4.94	37900	11400	2960	11400	1150
GLZLM_LGZE	0.00327	0.00175	0.00281	0.0015	0.00139	0.00329	0.00412	0.00201	0.00149	0.0013	0.00217
GLZLM_HGZE	1200	949	616	1010	1520	1510	418	749	1120	1160	964
GLZLM_SZLGE	0.003	0.00118	0.00145	0.000953	0.000771	0.0031	0.00256	0.0011	0.000769	0.000748	0.00139
GLZLM_SZHGE	824	473	290	712	820	1160	221	382	609	643	526
GLZLM_LZLGE	0.0137	16	14.3	0.015	5.26	0.00619	87.3	14.9	2.74	10	1.41
GLZLM_LZHGE	18900	20500000	3490000	8550	11900000	7330	16800000	8770000	3260000	13200000	971000
GLZLM_GLNU	20.4	148	129	290	119	21.2	168	451	123	230	212
GLZLM_ZLNU	236	902	609	3480	902	307	851	2540	884	1600	1980
GLZLM_ZP	0.478	0.0862	0.0808	0.502	0.109	0.605	0.0763	0.128	0.144	0.125	0.189

Patient	56	57	58	59	60	61	62	63	64	65	66
Type (grade1=1, grade2=2, grade3=3)	1	1	1	1	1	1	2	2	2	2	2
minValue	353	245	360	74	255	8	150	1790	833	128	67.3
meanValue	786	730	1050	908	913	1190	676	4470	3850	219	308
stdValue	64.9	77.6	169	155	133	153	91.5	659	1330	14.9	110
maxValue	1140	1130	2250	1720	1310	2250	1130	6370	6420	351	1010
HISTO_Skewness	-0.0668	-0.334	0.873	-0.401	-0.885	-0.759	-0.436	-0.146	-0.487	0.293	1.83
HISTO_Kurtosis	4.16	5.04	6.31	5.13	6.41	6.87	5.42	3.17	1.86	5.15	7.37
HISTO_Entropy_log10	1.33	1.35	1.35	1.38	1.48	1.23	1.37	1.58	1.68	1.24	1.38
HISTO_Energy	0.056	0.0579	0.0563	0.052	0.0414	0.0714	0.0524	0.031	0.0244	0.0701	0.0567
SHAPE_Volume (mL)	11.9	10.3	6.21	7.48	1.56	21.3	38.2	99.8	92.6	21.7	19.7
GLCM_Homogeneity	0.414	0.415	0.366	0.366	0.299	0.421	0.424	0.255	0.271	0.426	0.355
GLCM_Energy	0.00658	0.0073	0.00534	0.00479	0.00322	0.0081	0.0057	0.00151	0.00165	0.00801	0.00524
GLCM_Contrast	17.2	15.8	23.4	24.1	60.9	12.9	15.6	80	118	9.55	31.2
GLCM_Correlation	0.658	0.698	0.564	0.614	0.444	0.597	0.778	0.542	0.752	0.708	0.691
GLCM_Entropy_log10	2.41	2.39	2.48	2.53	2.69	2.28	2.48	2.98	3.09	2.26	2.57
GLCM_Dissimilarity	2.87	2.81	3.47	3.49	5.35	2.54	2.69	6.57	7.48	2.32	3.8
GLRLM_SRE	0.895	0.897	0.922	0.923	0.951	0.9	0.892	0.96	0.949	0.899	0.927
GLRLM_LRE	1.72	1.71	1.43	1.41	1.25	1.52	1.59	1.2	1.3	1.52	1.36
GLRLM_LGRE	0.000868	0.000916	0.00241	0.00118	0.00209	0.00103	0.00128	0.00131	0.00334	0.00163	0.00601
GLRLM_HGRE	1320	1300	605	1120	1710	1210	1260	1530	1450	734	350
GLRLM_SRLGE	0.000781	0.000833	0.00225	0.0011	0.00202	0.000947	0.00112	0.00127	0.00315	0.00148	0.00558
GLRLM_SRHGE	1180	1160	561	1040	1620	1090	1130	1460	1370	660	330
GLRLM_LRLGE	0.00146	0.00148	0.00334	0.0016	0.0025	0.00149	0.00209	0.00147	0.0047	0.00239	0.00812
GLRLM_LRHGE	2260	2250	844	1580	2120	1820	1980	1840	1920	1110	446
GLRLM_GLNU	3510	3120	1030	1150	202	1360	2120	219	259	1350	988
GLRLM_RLNU	50100	43800	15500	18700	4430	15000	31400	6420	9650	15200	15000
GLRLM_RP	0.856	0.859	0.896	0.899	0.935	0.867	0.855	0.946	0.929	0.867	0.902
NGLDM_Coarseness	0.000136	0.000151	0.000414	0.000364	0.00155	0.000437	0.000298	0.00121	0.0008	0.000447	0.000494
NGLDM_Contrast	0.0308	0.0326	0.0389	0.0454	0.114	0.0209	0.0302	0.185	0.58	0.0185	0.0805
NGLDM_Busyness	2.3	2	1.06	0.892	0.184	0.755	1.08	0.314	0.509	0.956	1.63
GLZLM_SZE	0.55	0.55	0.608	0.596	0.664	0.69	0.629	0.651	0.718	0.676	0.679
GLZLM_LZE	8380	7710	408	345	19.4	3980	5130	10.3	47	6280	619
GLZLM_LGZE	0.00105	0.00134	0.00284	0.00157	0.00252	0.00172	0.00169	0.00178	0.00276	0.00228	0.00623
GLZLM_HGZE	1380	1150	687	1130	1700	1170	1290	1510	1310	738	492
GLZLM_SZLGE	0.000647	0.000877	0.0018	0.00111	0.00171	0.00144	0.00103	0.00146	0.00177	0.0017	0.00419
GLZLM_SZHGE	761	599	438	667	1120	783	804	977	919	501	362
GLZLM_LZLGE	7.12	5.68	0.837	0.321	0.0217	3.42	4.8	0.00945	0.153	9.08	3.05
GLZLM_LZHGE	10000000	10500000	209000	390000	31700	4700000	5670000	15200	88200	4470000	136000
GLZLM_GLNU	397	342	210	236	72.4	168	270	103	109	156	188
GLZLM_ZLNU	2870	2720	1840	2050	922	1700	2850	1390	2510	1430	2350
GLZLM_ZP	0.134	0.147	0.254	0.246	0.424	0.171	0.159	0.471	0.449	0.148	0.275

Patient	67	68	69	70	71	72	73	74	75	76	77
Type (grade1=1, grade2=2, grade3=3)	2	2	2	2	2	2	2	2	2	2	2
minValue	532	634	2000	495	3040	147	164	2000	145	274	502
meanValue	1140	1180	4180	1230	4430	665	447	3400	420	528	1390
stdValue	143	139	649	122	431	93.2	69.8	445	74.4	110	254
maxValue	1870	1740	6580	2230	5770	1120	821	5500	636	1140	3110
HISTO_Skewness	0.0222	0.0709	0.0425	-0.374	0.132	0.524	-1.18	-0.0668	-0.0986	0.661	0.425
HISTO_Kurtosis	3.41	4.23	3.26	6.23	3.27	5.24	4.3	3.74	3.55	3.68	3.69
HISTO_Entropy_log10	1.45	1.51	1.56	1.24	1.6	1.37	1.34	1.51	1.59	1.5	1.4
HISTO_Energy	0.042	0.0389	0.0324	0.0759	0.0301	0.0568	0.0636	0.0386	0.0312	0.0359	0.0475
SHAPE_Volume (mL)	54.4	28.5	29	27.1	14.2	21.7	42.5	10.6	33.2	24.8	121
GLCM_Homogeneity	0.296	0.366	0.222	0.501	0.226	0.388	0.416	0.295	0.288	0.353	0.367
GLCM_Energy	0.00284	0.00419	0.00168	0.0135	0.00185	0.0057	0.0102	0.00288	0.00326	0.00311	0.00431
GLCM_Contrast	43.9	46.5	95.9	8.99	108	20.6	48.5	50	94.3	38.5	24.9
GLCM_Correlation	0.525	0.583	0.39	0.699	0.377	0.707	0.323	0.53	0.476	0.689	0.659
GLCM_Entropy_log10	2.75	2.68	2.9	2.13	2.85	2.52	2.33	2.73	2.92	2.71	2.6
GLCM_Dissimilarity	4.99	4.5	7.53	1.96	7.82	3.15	4.24	5.15	7.02	4.19	3.59
GLRLM_SRE	0.944	0.91	0.972	0.855	0.971	0.911	0.885	0.951	0.899	0.925	0.919
GLRLM_LRE	1.36	1.67	1.13	2.01	1.14	1.46	2.07	1.24	11.8	1.46	1.53
GLRLM_LGRE	0.0016	0.00169	0.00218	0.00161	0.00333	0.00101	0.00212	0.00394	0.00175	0.00575	0.00291
GLRLM_HGRE	927	1100	1050	778	1200	1230	816	745	1420	443	544
GLRLM_SRLGE	0.00152	0.00157	0.00214	0.00142	0.00328	0.000932	0.00194	0.00384	0.00162	0.00535	0.00269
GLRLM_SRHGE	876	1010	1020	663	1170	1130	711	709	1270	413	503
GLRLM_LRLGE	0.00211	0.00255	0.00236	0.00298	0.00354	0.00143	0.00385	0.00449	0.0244	0.00832	0.0044
GLRLM_LRHGE	1250	1810	1170	1570	1340	1760	1790	918	16700	630	801
GLRLM_GLNU	746	681	65.7	1640	43.3	1060	1010	101	1990	432	2070
GLRLM_RLNU	15800	14800	1900	16200	1350	15400	13400	2380	58700	10200	36600
GLRLM_RP	0.922	0.876	0.962	0.803	0.96	0.881	0.843	0.934	0.892	0.897	0.888
NGLDM_Coarseness	0.000493	0.000461	0.00333	0.000469	0.00509	0.000538	0.000336	0.00331	0.000103	0.000798	0.000204
NGLDM_Contrast	0.0772	0.11	0.235	0.0153	0.287	0.0389	0.0933	0.154	0.238	0.0985	0.0554
NGLDM_Busyness	0.843	0.833	0.136	0.81	0.0917	0.61	1.19	0.174	3.76	0.886	2.8
GLZLM_SZE	0.576	0.524	0.746	0.591	0.738	0.639	0.557	0.706	0.0908	0.584	0.587
GLZLM_LZE	35.4	409	4.67	13200	4.96	1850	2720	25.2	533	137	477
GLZLM_LGZE	0.00186	0.00244	0.00266	0.00276	0.00438	0.00128	0.00329	0.00585	0.0016	0.0061	0.0034
GLZLM_HGZE	955	1130	1050	732	1250	1320	695	753	1370	524	602
GLZLM_SZLGE	0.00115	0.00124	0.0023	0.00195	0.00395	0.000933	0.00217	0.00488	0.000138	0.00325	0.00207
GLZLM_SZHGE	557	586	790	438	950	846	386	543	108	332	366
GLZLM_LZLGE	0.0486	0.479	0.00754	16.9	0.0101	1.75	2.74	0.0475	0.716	0.589	1.38
GLZLM_LZHGE	30100	380000	4860	10400000	5330	1980000	2790000	18300	739000	52600	185000
GLZLM_GLNU	233	113	37.7	124	23.7	166	125	35.1	170	108	447
GLZLM_ZLNU	2030	1090	662	1050	460	1820	983	556	404	1110	3930
GLZLM_ZP	0.335	0.202	0.607	0.112	0.602	0.219	0.167	0.419	0.0841	0.257	0.244

Patient	78	79	80	81	82	83	84	85	86	87	88
Type (grade1=1, grade2=2, grade3=3)	2	2	2	2	2	2	2	2	2	2	2
minValue	388	347	142	102	249	383	407	61.1	243	242	220
meanValue	1110	642	883	680	541	980	898	669	534	507	552
stdValue	147	71.9	81.4	111	72.5	169	132	85.2	109	62.1	83.8
maxValue	1730	1240	1370	1160	871	1560	1230	1070	1150	842	885
HISTO_Skewness	0.483	0.381	-1.15	-0.0259	0.269	0.291	-0.946	-0.304	0.445	0.153	0.229
HISTO_Kurtosis	4.3	7.57	9.7	3.78	2.92	3.61	4.02	7.08	3.46	5.44	3.34
HISTO_Entropy_log10	1.44	1.3	1.21	1.44	1.48	1.57	1.57	1.32	1.49	1.41	1.51
HISTO_Energy	0.0452	0.0614	0.0765	0.0442	0.0378	0.0335	0.0352	0.0585	0.0372	0.0512	0.0363
SHAPE_Volume (mL)	26	17.1	53.2	32.4	18.3	54.5	22.8	11.4	23	33.9	4.56
GLCM_Homogeneity	0.366	0.369	0.452	0.371	0.333	0.327	0.33	0.463	0.315	0.375	0.342
GLCM_Energy	0.00447	0.00581	0.00968	0.00369	0.00258	0.00273	0.00368	0.00755	0.00301	0.00615	0.00276
GLCM_Contrast	32.1	19.5	9.96	20.1	26.6	55.3	88.8	10.1	59.4	38.3	28.5
GLCM_Correlation	0.663	0.564	0.686	0.771	0.754	0.641	0.465	0.793	0.488	0.469	0.773
GLCM_Entropy_log10	2.63	2.41	2.21	2.63	2.74	2.82	2.76	2.31	2.76	2.56	2.73
GLCM_Dissimilarity	4.04	3.23	2.2	3.24	3.84	5.17	6.33	2.15	5.43	4.04	3.85
GLRLM_SRE	0.917	0.929	0.881	0.919	0.935	0.928	0.912	0.88	0.933	0.91	0.936
GLRLM_LRE	1.64	1.36	1.66	1.39	1.31	1.44	1.79	1.68	1.46	2.4	1.31
GLRLM_LGRE	0.00133	0.0042	0.000761	0.000992	0.00137	0.0015	0.00157	0.00114	0.00435	0.0019	0.00146
GLRLM_HGRE	1270	496	1540	1300	994	1170	1600	1560	512	878	1130
GLRLM_SRLGE	0.00122	0.00394	0.000681	0.00092	0.00129	0.00141	0.00146	0.00104	0.00407	0.00176	0.00138
GLRLM_SRHGE	1170	462	1360	1200	932	1090	1460	1380	481	801	1060
GLRLM_LRLGE	0.00211	0.00558	0.0012	0.00134	0.00178	0.00204	0.00255	0.00167	0.00639	0.00432	0.00184
GLRLM_LRHGE	2070	667	2570	1810	1280	1700	2870	2600	714	2020	1440
GLRLM_GLNU	824	410	3940	1390	677	621	706	632	568	1400	185
GLRLM_RLNU	15600	5690	38900	25900	15300	16000	17400	8060	13400	23800	4360
GLRLM_RP	0.885	0.906	0.842	0.893	0.913	0.904	0.88	0.84	0.906	0.878	0.914
NGLDM_Coarseness	0.000527	0.00124	0.00018	0.000392	0.000632	0.000441	0.000362	0.0011	0.000521	0.000274	0.00251
NGLDM_Contrast	0.057	0.0401	0.0127	0.0446	0.0757	0.159	0.227	0.0227	0.125	0.0651	0.0824
NGLDM_Busyness	0.636	0.478	1.36	0.85	0.721	0.95	0.893	0.258	1.31	1.4	0.17
GLZLM_SZE	0.51	0.619	0.632	0.607	0.623	0.555	0.443	0.695	0.566	0.54	0.649
GLZLM_LZE	169	176	20700	536	104	73.4	230	3230	80.5	359	99.9
GLZLM_LGZE	0.00146	0.00482	0.00121	0.00121	0.0015	0.00192	0.00222	0.00228	0.0045	0.00215	0.00192
GLZLM_HGZE	1320	519	1460	1300	1070	1140	1570	1610	609	954	1260
GLZLM_SZLGE	0.000709	0.00288	0.000879	0.000726	0.00101	0.00122	0.00114	0.00145	0.00247	0.0011	0.0015
GLZLM_SZHGE	685	333	900	775	684	621	683	1100	377	535	842
GLZLM_LZLGE	0.164	0.427	13.3	0.469	0.13	0.0782	0.2	2.21	0.338	0.501	0.13
GLZLM_LZHGE	205000	83100	32500000	643000	96100	85800	374000	4850000	29500	279000	91200
GLZLM_GLNU	149	93.4	320	266	192	166	109	64.3	154	229	56
GLZLM_ZLNU	1060	738	2800	2720	2090	1670	771	785	1450	1900	697
GLZLM_ZP	0.202	0.276	0.12	0.223	0.293	0.277	0.171	0.134	0.282	0.212	0.317

Patient	89	90	91	92	93	94	95	96	97	98	99
Type (grade1=1, grade2=2, grade3=3)	2	2	2	2	2	2	2	2	2	2	2
minValue	897	476	71.2	180	266	155	84.8	370	180	305	25.9
meanValue	5000	1220	492	614	980	572	655	763	727	1080	647
stdValue	614	75.2	137	86.4	88.2	84.7	170	91.8	210	181	162
maxValue	7160	1480	948	1010	1680	1070	1470	1130	1660	2080	1340
HISTO_Skewness	-0.39	-2.61	-0.368	0.335	0.509	0.383	-0.126	0.185	-0.282	-0.288	-0.26
HISTO_Kurtosis	4.08	15.5	3.3	3.49	4.24	3.7	3.12	3.83	2.59	4.42	3.12
HISTO_Entropy_log10	1.4	1.17	1.59	1.42	1.2	1.38	1.49	1.49	1.55	1.42	1.5
HISTO_Energy	0.0474	0.0955	0.0309	0.045	0.0724	0.0491	0.0378	0.0404	0.0322	0.0494	0.0382
SHAPE_Volume (mL)	50.1	88.8	40.3	38.9	30.5	64.8	170	31.5	97.1	98.6	43
GLCM_Homogeneity	0.358	0.483	0.347	0.465	0.465	0.425	0.465	0.352	0.376	0.401	0.431
GLCM_Energy	0.00446	0.0193	0.00224	0.00591	0.00947	0.00522	0.00458	0.0038	0.00303	0.00559	0.0041
GLCM_Contrast	34.5	18	29.8	9.68	7.13	12.8	14	43.1	24.6	19.2	14.6
GLCM_Correlation	0.536	0.438	0.852	0.884	0.766	0.808	0.889	0.62	0.85	0.772	0.875
GLCM_Entropy_log10	2.59	2.03	2.87	2.44	2.17	2.47	2.58	2.71	2.74	2.54	2.61
GLCM_Dissimilarity	4.09	2.51	3.9	2.13	1.97	2.54	2.4	4.29	3.41	3.07	2.63
GLRLM_SRE	0.92	0.84	0.926	0.873	0.876	0.893	0.864	0.925	0.916	0.903	0.895
GLRLM_LRE	1.54	3.03	1.37	1.73	1.68	1.58	1.88	1.52	1.53	1.65	1.58
GLRLM_LGRE	0.000669	0.000627	0.00251	0.00108	0.00101	0.00136	0.00204	0.00151	0.00484	0.00194	0.00161
GLRLM_HGRE	1840	2240	1080	1210	1090	923	797	1200	665	848	1020
GLRLM_SRLGE	0.000622	0.000557	0.00226	0.000951	0.000887	0.00122	0.00174	0.00143	0.00439	0.00176	0.00146
GLRLM_SRHGE	1690	1870	1000	1070	965	832	697	1120	606	764	924
GLRLM_LRLGE	0.000984	0.00151	0.00398	0.00179	0.0017	0.00217	0.00408	0.00208	0.00764	0.00307	0.00247
GLRLM_LRHGE	2820	7010	1460	2030	1800	1410	1420	1780	1040	1400	1570
GLRLM_GLNU	1100	4660	990	1420	1930	2750	7640	523	991	2410	1430
GLRLM_RLNU	19600	39100	26800	23400	19600	42900	145000	11200	25500	40300	29000
GLRLM_RP	0.889	0.793	0.9	0.831	0.837	0.857	0.814	0.897	0.884	0.868	0.858
NGLDM_Coarseness	0.000336	0.000105	0.000482	0.0005	0.000399	0.00022	0.0000847	0.000714	0.000499	0.000209	0.000425
NGLDM_Contrast	0.0653	0.0234	0.116	0.0301	0.0159	0.0297	0.0428	0.0896	0.0987	0.0427	0.0506
NGLDM_Busyness	0.876	1.87	0.998	0.701	1.03	1.77	5.31	0.511	1.18	1.88	0.935
GLZLM_SZE	0.566	0.527	0.629	0.641	0.627	0.639	0.605	0.612	0.596	0.54	0.636
GLZLM_LZE	246	52800	367	7310	16800	7670	29500	197	587	1970	978
GLZLM_LGZE	0.000891	0.00214	0.00249	0.00177	0.00128	0.00157	0.00192	0.00228	0.00423	0.00225	0.00193
GLZLM_HGZE	1800	1810	1050	1310	1260	1070	906	1280	641	834	1130
GLZLM_SZLGE	0.000621	0.00185	0.00153	0.0014	0.000959	0.00105	0.00116	0.00163	0.00227	0.00122	0.00113
GLZLM_SZHGE	995	865	647	817	791	679	553	788	400	449	723
GLZLM_LZLGE	0.148	22.2	0.924	7.43	17.2	9.67	48.4	0.206	1.7	2.52	1.19
GLZLM_LZHGE	424000	126000000	330000	7560000	16800000	6450000	22700000	201000	521000	1590000	970000
GLZLM_GLNU	236	176	260	135	149	325	978	124	232	373	243
GLZLM_ZLNU	1830	1300	3640	1730	1080	3620	10800	1420	2580	2860	3110
GLZLM_ZP	0.23	0.0749	0.275	0.116	0.0915	0.145	0.126	0.276	0.221	0.179	0.187

Patient	100	101	102	103	104	105	106	107	108	109	110
Type (grade1=1, grade2=2, grade3=3)	2	2	2	2	2	2	2	2	2	2	2
minValue	230	37.6	79.8	333	206	533	2380	160	1790	266	281
meanValue	1100	504	413	792	660	1320	3970	560	3460	478	555
stdValue	143	111	34.8	107	73.3	165	443	81.7	489	70.3	59
maxValue	1920	928	705	1440	1080	1750	5420	841	4880	638	760
HISTO_Skewness	0.95	-0.402	0.319	1.94	-0.31	-0.386	-0.13	0.331	-0.569	0.0272	-0.664
HISTO_Kurtosis	6.32	4.36	7.87	8.88	4.61	2.79	3.33	3.04	3.01	2.14	3.79
HISTO_Entropy_log10	1.31	1.5	1.14	1.31	1.34	1.54	1.58	1.49	1.59	1.65	1.49
HISTO_Energy	0.065	0.0396	0.0872	0.0635	0.0559	0.0326	0.0316	0.0384	0.0299	0.0256	0.0397
SHAPE_Volume (mL)	83	10.2	34.4	21	79.5	16	26.7	11.2	16.6	4.59	73.4
GLCM_Homogeneity	0.505	0.326	0.505	0.481	0.426	0.318	0.281	0.377	0.279	0.207	0.352
GLCM_Energy	0.0138	0.00283	0.0138	0.00978	0.00602	0.00356	0.00201	0.00326	0.00191	0.00295	0.00398
GLCM_Contrast	13.4	37.2	6.1	11.7	13.8	97	86.9	18.6	75.3	313	39.1
GLCM_Correlation	0.726	0.694	0.72	0.842	0.745	0.381	0.442	0.842	0.595	-0.0369	0.609
GLCM_Entropy_log10	2.23	2.78	2.04	2.28	2.43	2.83	2.91	2.66	2.9	2.77	2.66
GLCM_Dissimilarity	2.33	4.31	1.71	2.18	2.56	6.8	6.5	3.14	6.18	14.6	4.31
GLRLM_SRE	0.816	0.938	0.852	0.863	0.893	0.892	0.949	0.92	0.952	0.933	0.919
GLRLM_LRE	4.85	1.29	1.87	1.84	1.57	6.13	1.26	1.4	1.23	2.13	1.51
GLRLM_LGRE	0.00103	0.00189	0.000911	0.00164	0.00105	0.00072	0.00201	0.000908	0.00224	0.00167	0.00109
GLRLM_HGRE	1150	1220	1210	789	1180	1840	1230	1520	1310	1500	1420
GLRLM_SRLGE	0.000845	0.00177	0.000783	0.00141	0.000956	0.000644	0.00194	0.000842	0.00219	0.00157	0.00103
GLRLM_SRHGE	939	1140	1040	697	1050	1640	1170	1400	1240	1400	1300
GLRLM_LRLGE	0.00477	0.00241	0.00166	0.00306	0.00158	0.00395	0.00238	0.00124	0.00247	0.00295	0.00148
GLRLM_LRHGE	5420	1570	2250	1340	1860	11800	1570	2090	1670	3270	2200
GLRLM_GLNU	10800	390	2500	1030	3870	2610	267	382	114	169	1280
GLRLM_RLNU	130000	8580	20200	12000	53500	71100	7590	8150	3450	6100	27700
GLRLM_RP	0.756	0.917	0.806	0.814	0.858	0.871	0.932	0.893	0.935	0.917	0.891
NGLDM_Coarseness	0.0000469	0.00111	0.000311	0.000683	0.000161	0.000076	0.000839	0.00134	0.00215	0.000475	0.000286
NGLDM_Contrast	0.026	0.0841	0.00935	0.0311	0.024	0.188	0.223	0.0661	0.25	0.829	0.0931
NGLDM_Busyness	6.76	0.318	0.983	0.653	1.91	4.06	0.494	0.284	0.189	0.902	1.11
GLZLM_SZE	0.452	0.665	0.703	0.651	0.661	0.217	0.635	0.626	0.701	0.435	0.593
GLZLM_LZE	179000	104	24800	5980	9170	643	23	291	20.9	49.7	415
GLZLM_LGZE	0.00139	0.0018	0.00128	0.00163	0.00165	0.00101	0.00259	0.00126	0.0034	0.00238	0.00179
GLZLM_HGZE	1080	1260	1290	1090	1140	1670	1190	1600	1180	1430	1210
GLZLM_SZLGE	0.000639	0.000953	0.00102	0.000949	0.00128	0.000352	0.00177	0.000982	0.00292	0.00113	0.00133
GLZLM_SZHGE	463	859	912	714	737	307	740	997	807	590	685
GLZLM_LZLGE	177	0.105	21.1	10.6	7.88	0.383	0.025	0.255	0.0175	0.0484	0.266
GLZLM_LZHGE	183000000	125000	29600000	3500000	10900000	1250000	30700	364000	36100	83900	667000
GLZLM_GLNU	383	109	159	101	457	289	102	88.3	48.8	43	282
GLZLM_ZLNU	2090	1510	1590	1150	4990	854	1390	917	868	383	3030
GLZLM_ZP	0.0449	0.336	0.0952	0.139	0.15	0.101	0.404	0.225	0.458	0.255	0.246

Patient	111	112	113	114	115	116	117	118	119	120	121
Type (grade1=1, grade2=2, grade3=3)	2	2	2	2	2	2	2	2	2	2	2
minValue	358	141	348	153	122	1280	1580	349	1060	227	1190
meanValue	890	538	864	427	252	4410	4740	638	3920	448	3080
stdValue	115	76.6	107	64.7	50.7	696	1090	50.2	654	75.1	387
maxValue	1380	837	1270	815	489	6550	8530	865	5660	816	5130
HISTO_Skewness	-0.00248	0.253	-0.262	0.796	0.603	-0.0873	-0.468	-0.319	-0.561	0.385	-0.218
HISTO_Kurtosis	3.84	5.24	3.02	6.76	3.48	2.5	3.17	3.9	3.94	3.38	4.03
HISTO_Entropy_log10	1.47	1.43	1.49	1.35	1.54	1.52	1.59	1.4	1.56	1.51	1.4
HISTO_Energy	0.0424	0.0479	0.0378	0.0628	0.0335	0.0341	0.0318	0.0465	0.0325	0.0359	0.0491
SHAPE_Volume (mL)	108	32.1	96.8	12.7	31.9	82.5	176	9.64	139	4.06	64.4
GLCM_Homogeneity	0.407	0.324	0.367	0.4	0.292	0.32	0.284	0.335	0.327	0.32	0.334
GLCM_Energy	0.00549	0.00387	0.00375	0.00766	0.00285	0.00261	0.00184	0.0034	0.00321	0.00246	0.00449
GLCM_Contrast	27.6	50.6	30	20.9	62.4	45.6	73.9	24.4	50.3	28.1	41.2
GLCM_Correlation	0.699	0.434	0.71	0.666	0.601	0.646	0.646	0.647	0.634	0.778	0.402
GLCM_Entropy_log10	2.59	2.65	2.66	2.44	2.82	2.77	2.97	2.61	2.82	2.75	2.59
GLCM_Dissimilarity	3.53	4.8	3.93	3.09	5.93	4.77	6.03	3.72	5.04	4.02	4.51
GLRLM_SRE	0.882	0.94	0.905	0.91	0.93	0.94	0.948	0.936	0.907	0.943	0.934
GLRLM_LRE	2.61	1.32	1.72	1.48	1.78	1.33	1.26	1.3	3.67	1.26	1.4
GLRLM_LGRE	0.00113	0.00124	0.000937	0.00196	0.00355	0.000945	0.00286	0.000995	0.0015	0.00308	0.00136
GLRLM_HGRE	1200	1420	1370	772	619	1560	980	1350	1690	669	1020
GLRLM_SRLGE	0.00102	0.00119	0.000857	0.00181	0.0033	0.0009	0.00268	0.000945	0.00141	0.00293	0.00128
GLRLM_SRHGE	1060	1330	1240	709	578	1460	929	1260	1520	632	948
GLRLM_LRLGE	0.00277	0.00149	0.00154	0.00272	0.0064	0.0012	0.00393	0.00123	0.00548	0.00375	0.00183
GLRLM_LRHGE	3080	1850	2390	1110	1060	2070	1230	1770	6290	836	1430
GLRLM_GLNU	3500	300	2710	759	561	275	452	445	2250	137	773
GLRLM_RLNU	68200	5540	59500	10200	15000	7080	12600	8180	61000	3310	13900
GLRLM_RP	0.844	0.919	0.873	0.878	0.908	0.919	0.93	0.916	0.887	0.925	0.911
NGLDM_Coarseness	0.000111	0.00113	0.000126	0.000754	0.00039	0.0011	0.000613	0.00101	0.000129	0.00336	0.000463
NGLDM_Contrast	0.0658	0.089	0.0879	0.0404	0.193	0.133	0.203	0.0566	0.147	0.0873	0.0721
NGLDM_Busyness	3.14	0.275	2.93	0.527	1.75	0.335	0.716	0.349	2.56	0.178	0.821
GLZLM_SZE	0.455	0.662	0.503	0.685	0.494	0.626	0.681	0.638	0.338	0.611	0.611
GLZLM_LZE	3260	51.5	441	1440	54.2	46.8	33.7	111	322	38.7	78.8
GLZLM_LGZE	0.00154	0.00192	0.00119	0.00274	0.00349	0.00123	0.00262	0.00139	0.0015	0.00387	0.0016
GLZLM_HGZE	1200	1430	1300	904	689	1540	968	1290	1550	706	994
GLZLM_SZLGE	0.000747	0.00164	0.000696	0.0021	0.00164	0.000933	0.00184	0.00105	0.000387	0.00277	0.000968
GLZLM_SZHGE	554	928	638	642	362	949	656	819	485	451	608
GLZLM_LZLGE	2.98	0.0445	0.346	2.17	0.19	0.0371	0.207	0.0762	0.251	0.09	0.0808
GLZLM_LZHGE	3690000	64000	633000	972000	28000	70400	28900	172000	580000	23800	82100
GLZLM_GLNU	386	88.6	477	109	148	96.1	177	119	348	47	209
GLZLM_ZLNU	2720	1030	3460	1540	1150	1150	2720	1190	1770	503	1960
GLZLM_ZP	0.13	0.363	0.172	0.243	0.26	0.357	0.412	0.301	0.156	0.349	0.317

Patient	122	123	124	125	126	127	128	129	130	131	132
Type (grade1=1, grade2=2, grade3=3)	3	3	3	3	3	3	3	3	3	3	3
minValue	1260	1570	320	149	139	295	25	219	122	250	120
meanValue	1940	5640	764	461	419	1160	239	521	353	812	546
stdValue	184	1090	147	105	74.4	351	91.3	124	93.7	183	115
maxValue	3720	9340	1360	807	1310	2450	691	1030	878	1360	993
HISTO_Skewness	1.24	-0.762	-0.0223	0.45	1.67	0.375	1.95	0.406	1.19	-0.592	-0.487
HISTO_Kurtosis	9.14	3.35	3.49	3.2	12.3	2.62	6.28	2.76	6.44	2.76	3.37
HISTO_Entropy_log10	1.27	1.54	1.56	1.61	1.17	1.62	1.28	1.59	1.47	1.6	1.53
HISTO_Energy	0.0662	0.0344	0.0331	0.0288	0.0879	0.027	0.0843	0.029	0.0415	0.0298	0.0355
SHAPE_Volume (mL)	26.6	71.8	4.92	12.3	144	58.6	5.88	14.1	21	59.8	1.63
GLCM_Homogeneity	0.311	0.265	0.293	0.239	0.444	0.263	0.385	0.268	0.306	0.3	0.329
GLCM_Energy	0.00511	0.00187	0.00195	0.00153	0.0135	0.0015	0.0134	0.00167	0.00323	0.0024	0.00278
GLCM_Contrast	32.4	85.3	43.2	135	17.6	120	145	94.8	49.4	109	44.9
GLCM_Correlation	0.228	0.451	0.72	0.368	0.434	0.452	0.305	0.483	0.408	0.476	0.644
GLCM_Entropy_log10	2.45	2.93	2.86	3.01	2.18	3.04	2.36	2.96	2.69	2.93	2.78
GLCM_Dissimilarity	4.27	6.61	4.88	8.37	2.77	7.81	7.4	6.87	5.14	6.94	4.67
GLRLM_SRE	0.952	0.956	0.953	0.963	0.872	0.949	0.896	0.955	0.946	0.936	0.941
GLRLM_LRE	1.22	1.22	1.21	1.2	2.14	1.33	2.15	1.24	1.33	1.46	1.36
GLRLM_LGRE	0.00381	0.00159	0.00304	0.00183	0.00507	0.0033	0.00325	0.00402	0.00491	0.00199	0.00214
GLRLM_HGRE	363	1230	855	1060	273	800	561	688	469	1180	1070
GLRLM_SRLGE	0.00364	0.00154	0.00293	0.00177	0.00445	0.0031	0.0029	0.00383	0.00465	0.00188	0.00203
GLRLM_SRHGE	348	1170	815	1020	241	762	522	661	446	1100	1000
GLRLM_LRLGE	0.00458	0.00184	0.00349	0.00216	0.0107	0.00473	0.00725	0.00513	0.00646	0.00274	0.00271
GLRLM_LRHGE	435	1530	1030	1260	544	1030	942	833	610	1780	1480
GLRLM_GLNU	493	639	163	241	6920	1010	277	274	578	1200	325
GLRLM_RLNU	6690	16800	4410	7710	63500	33200	2980	8580	12400	35700	8100
GLRLM_RP	0.937	0.94	0.938	0.948	0.825	0.928	0.85	0.939	0.924	0.908	0.916
NGLDM_Coarseness	0.000833	0.000368	0.00228	0.000772	0.0000739	0.000177	0.000733	0.000836	0.000552	0.000191	0.00107
NGLDM_Contrast	0.0483	0.216	0.122	0.296	0.0165	0.307	0.209	0.236	0.1	0.266	0.109
NGLDM_Busyness	0.898	1.07	0.212	0.643	8.2	3.57	0.97	0.81	1.13	2.04	0.37
GLZLM_SZE	0.67	0.679	0.664	0.669	0.536	0.617	0.643	0.636	0.584	0.621	0.628
GLZLM_LZE	18.6	18.6	19	9.68	30100	24.3	653	14.5	34.4	128	71.8
GLZLM_LGZE	0.00416	0.0019	0.00388	0.00205	0.00546	0.00274	0.00326	0.00369	0.00496	0.00244	0.00259
GLZLM_HGZE	401	1130	885	1110	381	859	921	756	517	1020	979
GLZLM_SZLGE	0.00296	0.00133	0.00296	0.00148	0.00258	0.00147	0.00237	0.00214	0.0026	0.0016	0.00148
GLZLM_SZHGE	286	738	611	763	227	541	641	499	325	602	594
GLZLM_LZLGE	0.0643	0.0173	0.0331	0.0152	131	0.0989	2.28	0.0594	0.13	0.133	0.0733
GLZLM_LZHGE	6060	26900	18000	9400	7150000	15200	194000	7870	14100	185000	85700
GLZLM_GLNU	173	273	65.8	114	493	414	26.4	119	188	351	106
GLZLM_ZLNU	1420	3870	926	1790	2990	5440	359	1650	1690	4970	1290
GLZLM_ZP	0.422	0.454	0.428	0.487	0.109	0.383	0.219	0.433	0.348	0.307	0.346

Patient	133	134	135	136	137	138	139	140	141	142	143
Type (grade1=1, grade2=2, grade3=3)	3	3	3	3	3	3	3	3	3	3	3
minValue	0	216	26	1090	367	334	24	902	305	539	105
meanValue	361	473	469	3070	805	744	518	3840	1110	977	335
stdValue	122	95.3	108	1370	74.5	104	284	624	195	160	82.6
maxValue	892	705	861	8890	1370	992	1470	6240	1780	1830	708
HISTO_Skewness	-0.433	-0.597	-0.0284	1.3	0.722	-0.47	0.266	-0.522	-0.511	0.586	0.306
HISTO_Kurtosis	3.22	2.89	2.97	3.64	6.81	3.14	1.92	4.17	3.78	3.52	3.31
HISTO_Entropy_log10	1.55	1.66	1.53	1.44	1.26	1.61	1.63	1.48	1.52	1.49	1.55
HISTO_Energy	0.0341	0.0259	0.0341	0.0591	0.0689	0.0283	0.0268	0.0419	0.0375	0.0405	0.0332
SHAPE_Volume (mL)	99.7	12.5	88	117	23.3	27.1	69.1	40.1	83.2	26.7	44.3
GLCM_Homogeneity	0.341	0.263	0.295	0.377	0.377	0.268	0.317	0.294	0.368	0.339	0.279
GLCM_Energy	0.00239	0.00162	0.00248	0.0103	0.00701	0.00189	0.0022	0.00286	0.00406	0.00435	0.00216
GLCM_Contrast	32.5	120	81.2	68.8	25.8	95.2	50.9	55.6	56.7	65.4	86.9
GLCM_Correlation	0.779	0.604	0.372	0.708	0.423	0.468	0.841	0.438	0.515	0.459	0.369
GLCM_Entropy_log10	2.83	3.01	2.85	2.56	2.39	2.94	2.97	2.76	2.72	2.68	2.89
GLCM_Dissimilarity	4.02	7.65	6.43	4.97	3.51	7.15	5.02	5.34	4.8	5.73	6.58
GLRLM_SRE	0.929	0.957	0.938	0.908	0.922	0.947	0.935	0.949	0.905	0.907	0.948
GLRLM_LRE	1.35	1.24	1.45	1.67	1.49	1.34	1.34	1.26	1.79	1.87	1.31
GLRLM_LGRE	0.00687	0.00464	0.0011	0.00842	0.00149	0.00137	0.0074	0.00163	0.00123	0.0041	0.00314
GLRLM_HGRE	772	1330	1260	440	846	1730	679	1330	1340	562	699
GLRLM_SRLGE	0.00617	0.00439	0.00104	0.00744	0.00139	0.00128	0.00665	0.00156	0.00115	0.00377	0.00299
GLRLM_SRHGE	715	1280	1180	419	782	1630	647	1260	1210	512	665
GLRLM_LRLGE	0.0108	0.00638	0.00157	0.0161	0.00215	0.00192	0.0117	0.00199	0.00196	0.00709	0.00409
GLRLM_LRHGE	1050	1600	1810	564	1240	2350	823	1690	2380	1000	900
GLRLM_GLNU	2900	218	2060	2440	1240	510	1980	508	1790	634	1030
GLRLM_RLNU	71400	7700	53400	37900	15400	16200	65400	10900	40500	13600	27900
GLRLM_RP	0.904	0.941	0.911	0.871	0.895	0.926	0.909	0.931	0.867	0.872	0.928
NGLDM_Coarseness	0.000146	0.00104	0.000111	0.000137	0.000362	0.000475	0.000163	0.000623	0.00016	0.000391	0.000232
NGLDM_Contrast	0.0948	0.351	0.159	0.225	0.0307	0.219	0.267	0.108	0.128	0.133	0.172
NGLDM_Busyness	3.2	0.419	3.48	6.92	1.09	0.719	5.23	0.561	2.1	1.52	2.26
GLZLM_SZE	0.651	0.671	0.576	0.662	0.552	0.577	0.682	0.646	0.582	0.503	0.618
GLZLM_LZE	383	17.5	66.1	6460	158	23.9	1700	29.7	936	355	25.9
GLZLM_LGZE	0.0062	0.0033	0.00121	0.00505	0.00177	0.00134	0.00499	0.00172	0.0019	0.00508	0.00318
GLZLM_HGZE	717	1420	1250	749	908	1650	800	1290	1250	647	752
GLZLM_SZLGE	0.00395	0.0019	0.000699	0.00304	0.00104	0.000708	0.00355	0.00116	0.00127	0.00243	0.00181
GLZLM_SZHGE	458	966	716	523	521	935	541	823	695	346	486
GLZLM_LZLGE	1.51	0.245	0.0641	77	0.208	0.0265	31.4	0.0306	0.834	1	0.0677
GLZLM_LZHGE	307000	15400	81600	570000	126000	44700	132000	41400	1110000	157000	16600
GLZLM_GLNU	756	104	589	332	235	179	756	179	306	108	372
GLZLM_ZLNU	10500	1760	5970	5690	1350	2160	13200	2040	3790	839	4530
GLZLM_ZP	0.281	0.467	0.287	0.26	0.228	0.353	0.36	0.398	0.209	0.188	0.376

Patient	144	145	146	147	148	149	150
Type (grade1=1, grade2=2, grade3=3)	3	3	3	3	3	3	3
minValue	18	22	2260	15	0	937	133
meanValue	478	705	4410	814	250	4380	320
stdValue	197	169	598	134	56.3	1230	68.7
maxValue	1490	1150	6400	1520	466	8120	751
HISTO_Skewness	0.218	-0.0138	-0.329	-1.05	0.0578	0.197	0.566
HISTO_Kurtosis	2.62	2.7	3.07	7.52	4.33	2.58	3.88
HISTO_Entropy_log10	1.52	1.59	1.57	1.29	1.49	1.64	1.45
HISTO_Energy	0.0331	0.0295	0.0316	0.0727	0.0397	0.0258	0.0417
SHAPE_Volume (mL)	66.7	4.84	18.8	102	12.1	54.2	40.9
GLCM_Homogeneity	0.353	0.297	0.238	0.393	0.282	0.254	0.32
GLCM_Energy	0.00316	0.00198	0.00158	0.00832	0.00272	0.00128	0.00378
GLCM_Contrast	54.6	61.9	103	24.7	67.3	111	54
GLCM_Correlation	0.656	0.626	0.392	0.528	0.349	0.506	0.422
GLCM_Entropy_log10	2.78	2.88	2.97	2.41	2.75	3.09	2.68
GLCM_Dissimilarity	5.03	5.63	7.45	3.25	5.86	7.46	5.34
GLRLM_SRE	0.917	0.948	0.966	0.908	0.955	0.957	0.927
GLRLM_LRE	1.86	1.31	1.16	1.48	1.25	1.21	1.58
GLRLM_LGRE	0.00491	0.00101	0.00172	0.00114	0.00302	0.00233	0.00454
GLRLM_HGRE	511	1640	1220	1230	1280	1090	449
GLRLM_SRLGE	0.00433	0.000962	0.00168	0.00106	0.0029	0.00225	0.0042
GLRLM_SRHGE	475	1560	1180	1110	1220	1050	419
GLRLM_LRLGE	0.0144	0.00127	0.0019	0.00155	0.00433	0.00272	0.0072
GLRLM_LRHGE	789	2110	1420	1830	1570	1320	684
GLRLM_GLNU	1440	214	190	6450	322	507	1100
GLRLM_RLNU	36000	6450	5560	74000	7370	17800	23100
GLRLM_RP	0.875	0.927	0.955	0.876	0.938	0.942	0.897
NGLDM_Coarseness	0.000189	0.00133	0.0012	0.000085	0.00102	0.000394	0.000227
NGLDM_Contrast	0.127	0.177	0.232	0.0371	0.0979	0.322	0.111
NGLDM_Busyness	3.38	0.335	0.363	3.38	0.339	1.39	2.95
GLZLM_SZE	0.572	0.627	0.694	0.709	0.639	0.683	0.574
GLZLM_LZE	711	28	6.85	17100	15.8	15	157
GLZLM_LGZE	0.00374	0.00129	0.00215	0.00192	0.00209	0.00279	0.00447
GLZLM_HGZE	584	1690	1200	1160	1320	1110	524
GLZLM_SZLGE	0.00203	0.001	0.00175	0.00156	0.0012	0.00203	0.00261
GLZLM_SZHGE	343	1050	823	801	861	745	319
GLZLM_LZLGE	9.48	0.0281	0.00825	13.6	0.0634	0.0258	0.635
GLZLM_LZHGE	124000	37900	8750	21600000	18500	14100	60100
GLZLM_GLNU	410	85.7	97.9	743	129	229	266
GLZLM_ZLNU	3540	1110	1540	10900	1420	4220	2310
GLZLM_ZP	0.234	0.386	0.542	0.218	0.429	0.466	0.252
