# Supplementary material for: The Diagnostic Value of Radiomics-Based Machine Learning in Predicting the Grade of Meningiomas Using Conventional Magnetic Resonance Imaging: A Preliminary Study
Source: Front Oncol. 2019 Dec 6;9:1338. doi: 10.3389/fonc.2019.01338 (PMC6908490; doi:10.3389/fonc.2019.01338)
Supplement: Supplement Material 2 — The definitions of texture analysis parameters. [file Data_Sheet_2.PDF]

Supplement material 2: The meaning of texture parameters

| Matrixes | Defination | TA feature name | Description |
|---|---|---|---|
| Histogram | the information derived from global histogram analysis | Skewness | Measures the asymmetry of the grey-level distribution in the histogram. |
| | | Kurtosis | Measures whether the grey-level distribution is peaked or flat relative to a normal distribution |
| | | Entropy | Measures the randomness of the distribution |
| | | Energy | Measures the uniformity of the distribution |
| Shape | | Volume | Volume of Interest in mL and in voxels. |
| Co-occurrence matrix (GLCM) | the arrangements of pairs of voxels to extract textural indices | Homogeneity | Homogeneity of grey-level voxel pairs |
| | | Energy | Uniformity of grey-level voxel pairs. |
| | | Correlation | Linear dependency of grey-levels in GLCM |
| | | Contrast | Local variations in the GLCM |
| | | Entropy | Randomness of grey-level voxel pairs |
| | | Dissimilarity | Variation of grey-level voxel pairs |
| Grey-Level Run Length Matrix (GLRLM) | the size of homogenous grey-level runs for each grey level | SRE | Distribution of the short homogeneous runs in an image |
| | | LRE | Distribution of the long homogeneous runs in an image |
| | | LGRE | Distribution of the low grey-level runs |
| | | HGRE | Distribution of the high grey-level runs |
| | | SRLGE | Distribution of the short homogenous runs with low grey-levels |
| | | SRHGE | Distribution of the short homogenous runs with high grey-levels |
| | | LRLGE | Distribution of the long homogeneous runs with low grey-levels |
| | | LRHGE | Distribution of the long homogeneous runs with high grey-levels |
| | | GLNUr | Non-uniformity of the grey-levels of the homogeneous runs |
| | | RLNU | Length of the homogeneous runs |
| | | RP | Homogeneity of the homogeneous runs |
| Neighbourhood Grey-Level Different Matrix (NGLDM) | the difference of grey-level between one voxel and its 26 neighbourhoods in three dimensions | Coarseness | Level of spatial rate of change in intensity |
| | | Contrast | Intensity difference between neighbouring regions |
| | | Busyness | Spatial frequency of changes in intensity |
| Grey-Level Zone Length Matrix (GLZLM) | the information on the size of homogenous zones for each grey-level in three dimensions | SZE | Distribution of the short homogeneous zones in an image |
| | | LZE | Distribution of the long homogeneous zones in an image |
| | | LGZE | Distribution of the low grey-level zones |
| | | HGZE | Distribution of the high grey-level zones |
| | | SZLGE | Distribution of the short homogenous zones with low grey-levels |
| | | SZHGE | Distribution of the short homogenous zones with high grey-levels |
| | | LZLGE | Distribution of the long homogenous zones with low grey-levels |
| | | LZHGE | Distribution of the long homogenous zones with high grey-levels |
| | | GLNUz | Non-uniformity of the grey-levels of the homogeneous zones |
| | | RLNU | Length of the homogeneous runs |
| | | ZP | Homogeneity of the homogeneous zones |
| | | Sphericity | Measures how spherical a volume of interest is |
| | | Compacity | Measures the degree to which the volume of interest is compact |
