# Supplementary material for: The Diagnostic Value of Radiomics-Based Machine Learning in Predicting the Grade of Meningiomas Using Conventional Magnetic Resonance Imaging: A Preliminary Study
Source: Front Oncol. 2019 Dec 6;9:1338. doi: 10.3389/fonc.2019.01338 (PMC6908490; doi:10.3389/fonc.2019.01338)
Supplement: Supplement Material 3 — The results of Mann-Whitney U-test in the examination of repeatability of texture analysis parameters extraction. [file Data_Sheet_3.PDF]

	Null Hypothesis	Significance (p value)			Decision
		WHO Grade I	WHO Grade II	WHO Grade III	
1	The distribution of minValue is the same across categories of Authors.	0.363	0.539	0.847	Retain the null hypothesis.
2	The distribution of meanValue is the same across categories of Authors.	0.593	0.782	0.290	Retain the null hypothesis.
3	The distribution of stdValue is the same across categories of Authors.	0.251	0.434	0.400	Retain the null hypothesis.
4	The distribution of maxValue is the same across categories of Authors.	0.316	0.365	0.186	Retain the null hypothesis.
5	The distribution of HISTO_Skewness is the same across categories of Authors.	0.569	0.782	0.813	Retain the null hypothesis.
6	The distribution of HISTO_Kurtosis is the same across categories of Authors.	0.313	0.529	0.914	Retain the null hypothesis.
7	The distribution of HISTO_Entropy_log10 is the same across categories of Authors.	0.334	0.438	0.621	Retain the null hypothesis.
8	The distribution of HISTO_Energy is the same across categories of Authors.	0.337	0.412	0.813	Retain the null hypothesis.
9	The distribution of SHAPE_Volume (mL) is the same across categories of Authors.	0.116	0.679	0.477	Retain the null hypothesis.
10	The distribution of GLCM_Homogeneity is the same across categories of Authors.	0.223	0.214	0.505	Retain the null hypothesis.
11	The distribution of GLCM_Energy is the same across categories of Authors.	0.784	0.476	0.451	Retain the null hypothesis.
12	The distribution of GLCM_Contrast is the same across categories of Authors.	0.134	0.342	0.591	Retain the null hypothesis.
13	The distribution of GLCM_Correlation is the same across categories of Authors.	0.063	0.462	0.533	Retain the null hypothesis.
14	The distribution of GLCM_Entropy_log10 is the same across categories of Authors.	0.665	0.311	0.561	Retain the null hypothesis.
15	The distribution of GLCM_Dissimilarity is the same across categories of Authors.	0.180	0.297	0.533	Retain the null hypothesis.
16	The distribution of GLRLM_SRE is the same across categories of Authors.	0.399	0.480	0.331	Retain the null hypothesis.
17	The distribution of GLRLM_LRE is the same across categories of Authors.	0.670	0.957	0.377	Retain the null hypothesis.
18	The distribution of GLRLM_LGRE is the same across categories of Authors.	0.121	0.104	0.354	Retain the null hypothesis.
19	The distribution of GLRLM_HGRE is the same across categories of Authors.	0.902	0.090	0.331	Retain the null hypothesis.
20	The distribution of GLRLM_SRLGE is the same across categories of Authors.	0.149	0.111	0.331	Retain the null hypothesis.
21	The distribution of GLRLM_SRHGE is the same across categories of Authors.	0.937	0.102	0.354	Retain the null hypothesis.
22	The distribution of GLRLM_LRLGE is the same across categories of Authors.	0.126	0.407	0.533	Retain the null hypothesis.
23	The distribution of GLRLM_LRHGE is the same across categories of Authors.	0.670	0.099	0.400	Retain the null hypothesis.
24	The distribution of GLRLM_GLNU is the same across categories of Authors.	0.729	0.591	0.400	Retain the null hypothesis.
25	The distribution of GLRLM_RLNU is the same across categories of Authors.	0.530	0.519	0.400	Retain the null hypothesis.
26	The distribution of GLRLM_RP is the same across categories of Authors.	0.449	0.326	0.377	Retain the null hypothesis.
27	The distribution of NGLDM_Coarseness is the same across categories of Authors.	0.226	0.921	0.533	Retain the null hypothesis.
28	The distribution of NGLDM_Contrast is the same across categories of Authors.	0.395	0.290	0.747	Retain the null hypothesis.
29	The distribution of NGLDM_Busyness is the same across categories of Authors.	0.292	0.866	0.715	Retain the null hypothesis.
30	The distribution of GLZLM_SZE is the same across categories of Authors.	0.806	0.933	0.715	Retain the null hypothesis.
31	The distribution of GLZLM_LZE is the same across categories of Authors.	0.458	0.293	0.310	Retain the null hypothesis.
32	The distribution of GLZLM_LGZE is the same across categories of Authors.	0.292	0.061	0.310	Retain the null hypothesis.
33	The distribution of GLZLM_HGZE is the same across categories of Authors.	0.649	0.231	0.290	Retain the null hypothesis.
34	The distribution of GLZLM_SZLGE is the same across categories of Authors.	0.834	0.075	0.354	Retain the null hypothesis.
35	The distribution of GLZLM_SZHGE is the same across categories of Authors.	0.665	0.645	0.310	Retain the null hypothesis.
36	The distribution of GLZLM_LZLGE is the same across categories of Authors.	0.692	0.137	0.747	Retain the null hypothesis.
37	The distribution of GLZLM_LZHGE is the same across categories of Authors.	0.348	0.529	0.270	Retain the null hypothesis.
38	The distribution of GLZLM_GLNU is the same across categories of Authors.	0.267	0.724	0.621	Retain the null hypothesis.
39	The distribution of GLZLM_ZLNU is the same across categories of Authors.	0.296	0.747	0.652	Retain the null hypothesis.
40	The distribution of GLZLM_ZP is the same across categories of Authors.	0.415	0.495	0.377	Retain the null hypothesis.
